# Fluorinated and *N*-Acryloyl-Modified 3,5-Di[(*E*)-benzylidene]piperidin-4-one Curcuminoids for the Treatment of Pancreatic Carcinoma

**DOI:** 10.3390/pharmaceutics15071921

**Published:** 2023-07-11

**Authors:** Hindole Ghosh, Sangita Bhattacharyya, Rainer Schobert, Prasad Dandawate, Bernhard Biersack

**Affiliations:** 1 Cancer Biology, University of Kansas Medical Center, 3901 Rainbow Boulevard, Kansas City, KS 66160, USA; hghosh@kumc.edu (H.G.); sbhattacharyya@kumc.edu (S.B.); pdandawate@kumc.edu (P.D.); 2 Organic Chemistry 1, University of Bayreuth, Universitaetsstrasse 30, 95440 Bayreuth, Germany; rainer.schobert@uni-bayreuth.de

**Keywords:** curcumin, EF24, acryl amide, anticancer agents, pancreatic carcinoma

## Abstract

Pancreatic carcinoma is a cancer disease with high mortality. Thus, new and efficient treatments for this disease are badly needed. Curcumin has previously shown promising effects in pancreatic cancer patients; however, this natural compound suffers from inadequate efficacy and bioavailability, preventing its clinical approval. The synthetic curcuminoid EF24 was developed with activities superior to curcumin against various cancer types. In this study, a series of analogs of EF24 were investigated for anticancer effects on pancreatic carcinoma models. A distinct activity boost was achieved by straightforward *N*-acrylation of EF24 analogs, in particular, of compounds bearing 3-fluoro-4-methoxybenzylidene, 3,4-difluorobenzylidene, and 4-trifluoromethylbenzylidene moieties, while no improvement was seen for *N*-acryloyl-modified EF24. Apoptosis induction and suppression of phospho-STAT3 levels were determined, the latter corroborated by docking of active curcuminoids into STAT3. Hence, promising new clues for the development of efficient and superior curcuminoids as valuable treatment options for one of the most lethal cancer diseases were discovered in this study.

## 1. Introduction

Pancreatic cancer is one of the most lethal human cancer diseases, and current treatment options include surgery, chemotherapy (e.g., gemcitabine, erlotinib, FOLFIRINOX), and radiotherapy [1,2]. Pancreatic ductal adenocarcinoma (PDAC) embodies an especially lethal form of pancreatic cancer, and the development of new potent drugs against PDAC turned out to be an unexpectedly great challenge [3]. Hence, prevention of PDAC outbreaks is an important issue, and in addition to the reduction of risk factors such as smoking, alcohol, and obesity, the chemoprevention of PDAC by dietary compounds (e.g., curcumin, flavonoids, retinoids, capsaicin) and synthetic drugs (e.g., cyclooxygenase/COX inhibitors, metformin, statin, gefitinib, histone deacetylase/HDAC inhibitors) can be a reasonable strategy to avoid PDAC formation [4]. Nevertheless, the quest for new drugs and drug targets to combat PDAC is of the highest priority.

**Figure 1:**
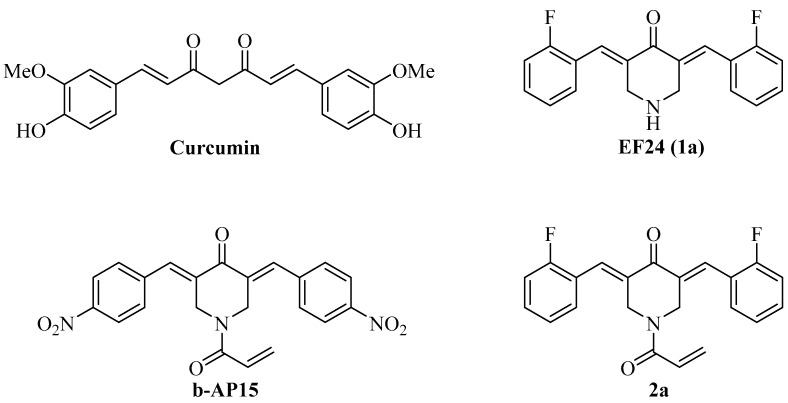
Curcumin and bioactive curcuminoids.

The pleiotropic anticancer activities of curcumin (diferuloylmethane), which is the main constituent of the spice turmeric (i.e., the roots of *Curcuma longa*), are well documented, and are especially promising to overcome cancer resistance (Figure 1) [5]. Its chemo-preventive and anticancer efficacies were demonstrated in various preclinical and clinical studies [6,7,8,9]. Curcumin was well tolerated in a phase II trial with advanced pancreatic cancer patients [9]. In addition to cancer, curcumin was also beneficial for the treatment of diabetes and the prevention of pregnancy complications, which highlights the general importance of this natural product for the treatment of human ailments [10,11].

Mechanistically, curcumin revealed versatile and relevant properties such as suppression/inhibition of Akt, nuclear factor-κB (NF-κB), signal transducer and activator of transcription-3 (STAT3), COX-2, and matrix metalloproteinase-2 (MMP-2) [12,13,14]. Moderate cytotoxicity and poor intestinal absorption hampered the advance of curcumin to clinical application until now [15,16]. Thus, (semi-)synthetic analogs of curcumin were developed having increased activity and bioavailability [17,18]. Bis(arylidene)acetones and their cyclic carbonyl analogs are of interest because of their significantly higher activities when compared with curcumin [8,18,19,20,21,22,23,24]. In particular, EF24 (**1a**), i.e., (3*E*,5*E*)-3,5-bis[(2-fluorophenyl)methylene]-4-piperidinone (Figure 1), was thoroughly investigated since it has shown considerable antimigratory, antiangiogenic, and antiproliferative activities, and because it interferes with crucial biochemical pathways in cancer cells [18,25,26,27,28,29,30,31,32]. Our groups also developed fluorinated piperidin-4-one-based curcuminoids with sound anticancer activities exceeding the potency of EF24 distinctly [33,34].

*N*-Acryloylation of piperidin-4-one-based curcuminoids led to the inhibition of deubiquitinases (DUBs) as a promising new anticancer mode of action of this compound class. Compound b-AP15 is a prominent example of an acryloyl-modified curcuminoid with DUB-inhibitory activity and the parent compound of the more recent derivative VLX1570 (Figure 1) [35,36,37]. The EF24-analog **2a** was also described as an anticancer active compound, albeit less active than its parent compound EF24 (Figure 1) [38].

Herein, we present some new fluorinated derivatives and *N*-acryloyl analogs of EF24 with promising anticancer activities against pancreatic carcinoma cells.

## 2. Materials and Methods

### 2.1. Chemistry

Starting compounds and reagents were purchased from Alfa Aesar (Karlsruhe, Germany), Merck (Darmstadt, Germany), and TCI (Zwijndrecht, Belgium). The known compounds **1a**, **1b**, **1d**, **1i**, **1j**, **2a**, **2d**, **2i**, and **2j** were prepared following the literature procedures, and their analytical data matched with the published data [38,39]. Column chromatography: silica gel 60 (230–400 mesh). Melting points (uncorrected), Electrothermal 9100; IR spectra, Perkin-Elmer Spectrum One FT-IR spectrophotometer with ATR sampling unit; NMR spectra, Bruker Avance 300/500 spectrometer; chemical shifts (δ) are given in parts per million (ppm) downfield from tetramethylsilane as the internal standard; Mass spectra, Thermo Finnigan MAT 8500 (EI), UPLC/Orbitrap (ESI).

***(3E,5E)-3,5-Bis-(3-fluoro-4-methoxybenzylidene)-piperidine-4-one (1c)***

Piperidin-4-one monohydrate hydrochloride (153 mg, 1.0 mmol) and 3-fluoro-4-methoxydehyde (308 mg, 2.0 mmol) were dissolved in MeOH (10 mL) and NaOH (40 mg) in H_2_O (1 mL) was added. The reaction mixture was stirred at room temperature for 2 h. The formed precipitate was collected, washed with MeOH/H_2_O, and dried in a vacuum. Yield: 110 mg (0.30 mmol, 30%); yellow solid of m.p. 198–199 °C; *υ*_max_(ATR)/cm^−1^ 3306, 2935, 2881, 2845, 1662, 1602, 1576, 1516, 1473, 1444, 1421, 1346, 1311, 1280, 1245, 1226, 1210, 1186, 1166, 1122, 1069, 1019, 997, 945, 921, 873, 840, 811, 761, 744, 728, 712, 670, 612, 597; ^1^H NMR (300 MHz, DMSO-d_6_) *δ* 3.89 (6 H, s), 3.9–4.0 (4 H, m), 7.2–7.4 (6 H, m), 7.50 (2 H, s); ^13^C NMR (75.5 MHz, DMSO-d_6_) *δ* 47.4, 56.1, 113.8, 115.2, 117.5–117.7 (m), 127.7–128.0 (m), 132.4, 135.1, 147.7–147.9 (m), 149.4, 152.7, 187.2; *m/z* (%) 371 (100) [M^+^], 343 (93), 204 (48), 178 (81), 164 (96), 149 (72), 121 (33), 101 (35).

***(3E,5E)-3,5-Bis-(2,3-difluorobenzylidene)-piperidine-4-one (1e)***

Piperidin-4-one monohydrate hydrochloride (153 mg, 1 mmol) and 2,5-difluorobenzaldehyde (284 mg, 2.0 mmol) were dissolved in methanol (10 mL) and NaOH (200 mg, dissolved in 2 mL H_2_O) was added. The reaction mixture was stirred at room temperature for 1 h. The formed precipitate was collected and dried in a vacuum. Yield: 190 mg (0.55 mmol, 55%); yellow solid of m.p. 224–225 °C; *υ*_max_(ATR)/cm^−1^ 3314, 2940, 2907, 2835, 1670, 1622, 1602, 1581, 1472, 1435, 1426, 1325, 1288, 1258, 1247, 1210, 1198, 1064, 1051, 1022, 998, 966, 947, 909, 883, 814, 788, 754, 744, 707, 600, 677, 642; ^1^H NMR (300 MHz, DMSO-d_6_) *δ* 2.8–2.9 (1 H, m), 3.88 (4 H, s), 7.2–7.3 (4 H, m), 7.4–7.6 (2 H, m), 7.61 (2 H, s); ^13^C NMR (75.5 MHz, DMSO-d_6_) *δ* 47.3, 118.1–118.3 (m), 124.7–125.0 (m), 126.2, 138.6, 146.2–151.5 (m), 187.0; *m/z* (%) 347 (47) [M^+^], 319 (77), 192 (35), 151 (100).

***(3E,5E)-3,5-Bis-(2,5-difluorobenzylidene)-piperidine-4-one (1f)***

Piperidin-4-one monohydrate hydrochloride (153 mg, 1 mmol) and 2,5-difluorobenzaldehyde (284 mg, 2.0 mmol) were dissolved in methanol (10 mL) and NaOH (200 mg, dissolved in 2 mL H_2_O) was added. The reaction mixture was stirred at room temperature for 1 h. The formed precipitate was collected and dried in a vacuum. Yield: 228 mg (0.66 mmol, 66%); yellow solid of m.p. 230 °C; *υ*_max_(ATR)/cm^−1^ 3316, 3083, 2940, 2829, 1679, 1635, 1621, 1605, 1587, 1478, 1427, 1316, 1281, 1244, 1202, 1193, 1177, 1147, 1091, 1006, 984, 966, 950, 929, 909, 876, 809, 797, 773, 744, 729, 707, 694, 678, 648, 602; ^1^H NMR (300 MHz, DMSO-d_6_) *δ* 2.7–2.8 (1 H, m), 3.90 (4 H, s), 7.3–7.4 (6 H, m), 7.55 (2 H, s); ^13^C NMR (75.5 MHz, DMSO-d_6_) *δ* 47.1, 116.7–117.9 (m), 123.7–124.0 (m), 124.9, 138.5, 154.9–159.4 (m), 187.0; *m/z* (%) 347 (52) [M^+^], 319 (76), 192 (36), 151 (100).

***(3E,5E)-3,5-Bis-(2,4,5-trifluorobenzylidene)-piperidine-4-one (1g)***

Piperidin-4-one monohydrate hydrochloride (77 mg, 0.5 mmol) and 2,4,5-trifluorobenzaldehyde (160 mg, 1.0 mmol) were dissolved in MeOH (5 mL). NaOH (40 mg, 1 mmol) and H_2_O (1 mL) were added and the reaction mixture was stirred at room temperature for 1 h. The formed precipitate was collected, washed with MeOH, and dried in a vacuum. Yield: 100 mg (0.26 mmol, 52%); yellow solid of m.p. 212–213 °C; *υ*_max_(ATR)/cm^−1^ 3317, 3090, 2937, 2831, 1678, 1626, 1603, 1493, 1425, 1337, 1304, 1238, 1226, 1201, 1160, 1107, 1017, 986, 959, 912, 891, 878, 859, 850, 762, 736, 707, 687; ^1^H NMR (300 MHz, CDCl_3_/DMSO-d_6_) *δ* 3.98 (4 H, s), 7.1–7.3 (4 H, m), 7.53 (2 H, s); ^13^C NMR (75.5 MHz, CDCl_3_/DMSO-d_6_) *δ* 47.1, 105.4, 105.7, 105.8, 106.0, 117.7, 118.0, 119.0, 119.2, 124.6, 127.9, 137.3, 144.2, 147.5, 151.3, 157.3, 186.1; *m/z* (%) 383 (16) [M^+^], 355 (49), 210 (31), 169 (100).

***(3E,5E)-3,5-Bis-(3-chloro-4-fluorobenzylidene)-piperidine-4-one (1h)***

Piperidin-4-one monohydrate hydrochloride (153 mg, 1.0 mmol) and 3-chloro-4-fluorobenzaldehyde (316 mg, 2.0 mmol) were dissolved in MeOH (10 mL) and NaOH (40 mg) in H_2_O (1 mL) was added. The reaction mixture was stirred at room temperature for 2 h. The formed precipitate was collected, washed with MeOH/H_2_O, and dried in a vacuum. Yield: 146 mg (0.38 mmol, 38%); yellow solid of m.p. 162–163 °C; *υ*_max_(ATR)/cm^−1^ 3315, 2953, 2834, 1656, 1594, 1574, 1497, 1462, 1402, 1292, 1269, 1243, 1186, 1135, 1060, 995, 940, 916, 879, 821, 778, 756, 741, 729, 711, 687, 666, 618; ^1^H NMR (300 MHz, DMSO-d_6_) *δ* 3.9–4.0 (4 H, m), 7.4–7.6 (6 H, m), 7.7–7.8 (2 H, m); C NMR (75.5 MHz, DMSO-d_6_) *δ* 47.2, 112.8, 117.0–117.2 (m), 119.7–119.9 (m), 131.0–131.4 (m), 132.3, 132.8, 136.8, 155.5, 158.8, 187.3; *m/z* (%) 381 (37) [M^+^], 379 (57) [M^+^], 351 (42), 168 (68), 133 (100).

***(3E,5E)-3,5-Bis-(3-fluorobenzylidene)-1-acryloylpiperidone (2b)***

Compound **1b** (61 mg, 0.20 mmol) was dissolved in acetone and treated with acryloyl chloride (30 µL, 0.37 mmol). K_2_CO_3_ (140 mg, 1.01 mmol, dissolved in 2 mL H_2_O) was added and the reaction mixture was stirred at room temperature for 24 h. Water (20 mL) was added and the formed precipitate was collected, washed with water, and dried in a vacuum. Yield: 30 mg (0.082 mmol, 41%); yellow solid of m.p. 119–120 °C; *υ*_max_(ATR)/cm^−1^ 3062, 1672, 1652, 1615, 1579, 1489, 1437, 1421, 1294, 1268, 1252, 1206, 1150, 1125, 1080, 1006, 993, 977, 947, 888, 872, 818, 789, 777, 758, 740, 687, 670, 610; ^1^H NMR (300 MHz, CDCl_3_) *δ* 4.7–5.0 (4 H, m), 5.59 (1 H, dd, J = 9.3 Hz, 2.9 Hz), 6.1–6.3 (2 H, m), 7.0–7.2 (6 H, m), 7.3–7.4 (2 H, m), 7.78 (2 H, s); ^13^C NMR (75.5 MHz, CDCl_3_) *δ* 116.5–116.8 (m), 126.1, 126.4, 129.2, 130.4, 130.5, 132.4, 136.4–136.4 (m), 161.1–164.4 (m), 165.5, 186.2; *m/z* (ESI, %) 366.2 (100) [M^+^], 135.1 (80).

***(3E,5E)-3,5-Bis-(3-fluoro-4-methoxybenzylidene)-1-acryloylpiperidone (2c)***

Compound **1c** (71 mg, 0.19 mmol) was dissolved in acetone and treated with acryloyl chloride (35 µL, 0.43 mmol). K_2_CO_3_ (197 mg, 1.43 mmol, dissolved in 2 mL H_2_O) was added and the reaction mixture was stirred at room temperature for 24 h. Water (20 mL) was added and the formed precipitate was collected, washed with water, and dried in a vacuum. Yield: 63 mg (0.15 mmol, 79%); yellow solid of m.p. 208 °C; *υ*_max_(ATR)/cm^−1^ 3075, 2979, 2939, 2843, 1671, 1646, 1599, 1573, 1517, 1456, 1439, 1424, 1364, 1309, 1294, 1286, 1266, 1247, 1234, 1218, 1176, 1134, 1121, 1060, 1018, 1000, 978, 962, 932, 918, 871, 857, 821, 790, 763, 737, 729, 711, 686, 664, 611, 599; ^1^H NMR (300 MHz, DMSO-d_6_) *δ* 3.91 (6 H, s), 4.9–5.0 (4 H, m), 5.6–5.7 (1 H, m), 6.0–6.1 (1 H, m), 6.5–6.6 (1 H, m), 7.3–7.4 (2 H, m), 7.4–7.6 (4 H, m), 7.6–7.7 (2 H, m); ^13^C NMR (75.5 MHz, DMSO-d_6_) *δ* 40.3, 46.4, 56.2, 113.9, 117.7–118.0 (m), 127.5, 128.2, 131.4, 135.0, 148.3–148.4 (m), 149.5, 152.8, 164.8, 185.6; *m/z* (%) 425 (96) [M^+^], 370 (100), 354 (92), 163 (27), 55 (43).

***(3E,5E)-3,5-Bis-(2,3-difluorobenzylidene)-1-acryloylpiperidone (2e)***

Compound **1e** (100 mg, 0.29 mmol) was suspended in acetone and treated with acryloyl chloride (53 µL, 0.66 mmol). K_2_CO_3_ (301 mg, 2.18 mmol, dissolved in 2 mL H_2_O) was added and the reaction mixture was stirred at room temperature for 24 h. Water (20 mL) was added and the formed precipitate was collected, washed with water, and dried in a vacuum. Yield: 95 mg (0.24 mmol, 83%); yellow solid of m.p. 159–161 °C; *υ*_max_(ATR)/cm^−1^ 3053, 3032, 2847, 1676, 1651, 1618, 1587, 1473, 1447, 1401, 1376, 1287, 1266, 1234, 1201, 1180, 1135, 1062, 1023, 1001, 979, 962, 937, 896, 839, 820, 797, 788, 776, 735, 706, 679, 646; ^1^H NMR (300 MHz, DMSO-d_6_) *δ* 4.7–4.9 (4 H, m), 5.59 (1 H, d, J = 12.7 Hz), 6.00 (1 H, d, J = 16.7 Hz), 6.5–6.6 (1 H, m), 7.3–7.4 (4 H, m), 7.5–7.6 (2 H, m), 7.70 (2 H, s); ^13^C NMR (75.5 MHz, DMSO-d_6_) *δ* 42.6, 46.3, 118.7–118.9 (m), 124.0, 125.1–125.2 (m), 126.2, 127.3, 128.3, 135.1, 146.3–151.6 (m), 164.9, 185.3; *m/z* (%) 401 (100) [M^+^], 346 (27), 326 (25), 151 (58), 55 (60).

***(3E,5E)-3,5-Bis-(2,5-difluorobenzylidene)-1-acryloylpiperidone (2f)***

Compound **1f** (100 mg, 0.29 mmol) was suspended in acetone and treated with acryloyl chloride (53 µL, 0.66 mmol). K_2_CO_3_ (301 mg, 2.18 mmol, dissolved in 2 mL H_2_O) was added and the reaction mixture was stirred at room temperature for 24 h. Water (20 mL) was added and the formed precipitate was collected, washed with water, and dried in a vacuum. Yield: 70 mg (0.17 mmol, 59%); yellow solid of m.p. 153–154 °C; *υ*_max_(ATR)/cm^−1^ 3089, 3003, 2860, 1678, 1652, 1615, 1588, 1485, 1462, 1441, 1428, 1394, 1344, 1326, 1302, 1277, 1266, 1256, 1227, 1196, 1179, 1130, 1091, 1054, 1029, 1004, 976, 967, 933, 874, 839, 815, 806, 788, 734, 710, 696, 680, 668, 608; ^1^H NMR (300 MHz, DMSO-d_6_) *δ* 4.7–4.9 (4 H, m), 5.61 (1 H, d, J = 12.7 Hz), 6.01 (1 H, d, J = 16.7 Hz), 6.6–6.7 (1 H, m), 7.3–7.5 (6 H, m), 7.65 (2 H, s); ^13^C NMR (75.5 MHz, DMSO-d_6_) *δ* 42.6, 46.2, 116.9–117.7 (m), 118.1–118.6 (m), 123.2, 127.4, 127.7, 128.3, 135.0, 154.9–159.5 (m), 165.0, 185.4; *m/z* (%) 401 (100) [M^+^], 346 (32), 151 (65), 55 (81).

***(3E,5E)-3,5-Bis-(2,4,5-trifluorobenzylidene)-1-acryloylpiperidone (2g)***

Compound **1g** (91 mg, 0.24 mmol) was suspended in acetone and treated with acryloyl chloride (30 µL, 0.37 mmol). K_2_CO_3_ (168 mg, 1.22 mmol, dissolved in 2 mL H_2_O) was added and the reaction mixture was stirred at room temperature for 24 h. Water (20 mL) was added and the formed precipitate was collected, washed with water, and dried in a vacuum. Yield: 50 mg (0.12 mmol, 50%); yellow solid of m.p. 118–119 °C; *υ*_max_(ATR)/cm^−1^ 3042, 1643, 1612, 1582, 1502, 1453, 1429, 1327, 1289, 1265, 1246, 1195, 1173, 1110, 1018, 975, 940, 876, 852, 799, 764, 750, 711, 698; ^1^H NMR (300 MHz, CDCl_3_) *δ* 4.6–4.8 (4 H, br s), 5.63 (1 H, dd, J = 3.0 Hz, 9.2 Hz), 6.2–6.3 (2 H, m), 7.0–7.1 (4 H, m), 7.73 (2 H, s); ^13^C NMR (75.5 MHz, CDCl_3_) *δ* 56.6, 106.2–106.5 (m), 118.1–118.4 (m), 126.2, 129.2, 129.4, 133.8, 145.2, 148.8, 152.3, 165.5, 185.1; *m/z* (%) 437 (96) [M^+^], 382 (46), 362 (25), 239 (23), 169 (77), 145 (22), 55 (100).

***(3E,5E)-3,5-Bis-(3-chloro-4-fluorobenzylidene)-1-acryloylpiperidone (2h)***

Compound **1h** (73 mg, 0.19 mmol) was dissolved in acetone and treated with acryloyl chloride (35 µL, 0.43 mmol). K_2_CO_3_ (197 mg, 1.43 mmol, dissolved in 2 mL H_2_O) was added and the reaction mixture was stirred at room temperature for 24 h. Water (20 mL) was added and the formed precipitate was collected, washed with water, and dried in a vacuum. Yield: 65 mg (0.15 mmol, 79%); yellow solid of m.p. 122–123 °C; *υ*_max_(ATR)/cm^−1^ 3033, 2848, 1675, 1639, 1615, 1582, 1497, 1463, 1441, 1403, 1361, 1342, 1262, 1244, 1226, 1207, 1196, 1176, 1127, 1062, 1027, 996, 977, 962, 943, 917, 899, 880, 828, 815, 790, 761, 728, 705, 691, 674, 653, 600; ^1^H NMR (300 MHz, DMSO-d_6_) *δ* 4.8–5.0 (4 H, m), 5.6–5.7 (1 H, m), 6.0–6.1 (1 H, m), 6.5–6.7 (1 H, m), 7.5–7.7 (6 H, m), 7.8–7.9 (2 H, m); ^13^C NMR (75.5 MHz, DMSO-d_6_) *δ* 42.7, 46.3, 109.2, 117.2–117.4 (m), 119.9–120.2 (m), 127.4, 128.4, 131.2, 132.5, 133.3, 134.0, 155.9, 159.2, 164.8, 185.8; *m/z* (%) 436 (63) [M^+^], 434 (100) [M^+^], 381 (36), 379 (61), 133 (47), 55 (68).

### 2.2. Anticancer Activity

#### 2.2.1. Cell Line and Culture Conditions

The pancreatic cancer cell lines MiaPaCa-2 and Panc-1 (American Type Culture Collection, Manassas, VA, USA), HPNE and THP-1 cells were a gift from Dr. Shrikant Anant’s lab. These cell lines were cultured in complete Dulbecco’s Modified Eagle Medium (DMEM). The complete media was prepared by mixing a heat-inactivated fetal bovine serum (10% concentration, FBS) (Sigma-Aldrich, St. Louis, MO, USA) and 1% antibiotic-antimycotic solution (Corning, Tewksbury, MA, USA) to DMEM media supplemented with 4.5 g/L of glucose, L-glutamine, and sodium pyruvate (Corning, MA, USA). All cell lines were cultured at 37 °C in a 5% CO_2_ humidifier and used within 15 passages.

#### 2.2.2. Proliferation Assay

A total of 5 × 10^3^ MiaPaCa-2 and Panc-1 cells/well were added in 96-well plates. Post 24 h of plating, the cells were treated with increasing concentrations of curcuminoids (0–10 μM concentration). An enzymatic hexosaminidase assay [40] was used to record the viability of pancreatic cancer cells at different time points. For THP-1 cells, a CCK-8 assay (Abcam#ab228554, Burlingame, CA, USA) was used. Briefly, 3 × 10^3^ were plated and treated with curcuminoids after 24 h of plating. The CCK8 regent was incubated with THP1 cells for 60 min at 37 °C, and the absorbance (OD value) was recorded at 450 nm [41]. The percent inhibition of proliferation was estimated by comparing the treated groups’ cell viability to the untreated cells.

#### 2.2.3. Caspase 3/7 Assay

To study the caspase3/7 activity in pancreatic cancer cells (MiaPaCa-2 and Panc-1) after EF24 and **2d** treatments, we used the Apo-one Homogeneous Caspase-3/7 Assay kit (Promega Corporation, Madison, WI, USA). We followed the manufacturer’s instructions to perform this assay.

#### 2.2.4. Western Blot

A total of 5 × 10^5^ MiaPaCa-2 and Panc-1 were added in 10 cm dishes for Western blot analysis. Cells were treated with vehicle, **2d**, and EF24 (at IC_50_ concentrations) after 24 h of plating. After 48 h of the treatment, the media was aspirated, washed with PBS, and the cells were lysed and sonicated in a lysis buffer + protease inhibitor cocktail (ThermoScientific, Rockford, IL, USA). The cell lysates were further centrifuged at 6000 rpm for 10 min in the cool centrifuge. The total protein levels were calculated by using a BCA method (ThermoScientific, IL, USA). Equal amounts of protein were loaded into the gels for separation using gel electrophoresis. The separated proteins on the gel were then transferred onto PVDF membranes (Immobilon, Millipore, Bedford, MA, USA) for 2 h at 90 V. The membranes were removed from the transfer assembly, incubated in 5% skimmed milk for 1 h, and washed with TBST thrice for 5 min. Finally, these membranes were probed with primary antibodies and kept overnight at 4 °C on a shaker. The next day, the blots were washed, probed with the respective secondary antibodies for 1 h, and rewashed before identification. The chemiluminescence system reagents (GE Health Care, Piscataway, NJ, USA) were used to develop the blots. The ChemiDoc-XRS+ instrument (Biorad) was used to record the protein levels and image lab software was used to quantify the protein levels. BCL2 (CST#4223), Bax (CST#2772), BCL-XL (CST#2762), PARP (CST#9542), and p-STAT3 (CST#4113s) antibodies were obtained from CST (Cell Signaling Technology, Beverly, MA, USA), and β-actin (sc-47778) was purchased from Santa Cruz Biotech, Inc. (Santa Cruz, CA, USA). The antibodies were diluted at a 1:1000 dilution in 5% BSA in TBST.

#### 2.2.5. Molecular Docking

The molecular docking was executed using the Autodock Vina 1.1.2 software program to study the interaction of active curcuminoids with the STAT3 protein (PDB ID: 6NJS) [42,43]. This pdb has a high resolution of 2.70 Å and a co-crystallized ligand and is ideal for studying STAT3 inhibitors [44]. The 3D grid was designed around the SH2 domain containing interacting amino acids with a co-crystallized ligand. A grid box was designed for docking using a grid center spacing of 1.0 Å and a 56 × 82 × 68-point size. The curcuminoid ligands and STAT3 protein preparation was conducted by using the default parameters of Autodock vina tools 1.5.7 and total Kollman and Gasteiger charges were added to the STAT3 proteins. We used the Lamarckian generic algorithm to calculate the STAT3–curcuminoid conformations. We evaluated ten predicted conformations of the curcuminoid–STAT3 complex, and the most stable predicted conformation based on the binding energy and hydrogen bonds. The curcuminoid–STAT3 complexes were evaluated and visualized using the educational Pymol program (https://pymol.org/2/ (accessed on 14 April 2023)) [45].

#### 2.2.6. Statistical Analysis

All data values are given as mean ± standard deviation (SD). Experimental data were examined in comparison with the control group by using an unpaired two-tailed t-test and one-way ANOVA.

## 3. Results

**Scheme 1:**
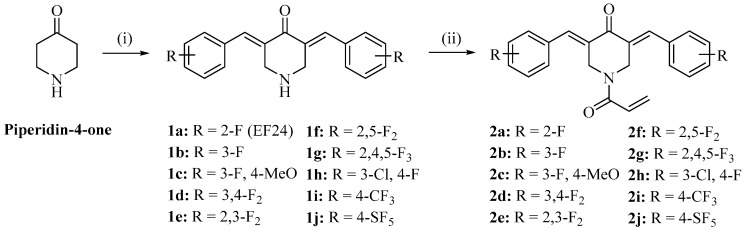
Reagents and conditions: (i) aryl aldehyde, NaOH, MeOH/H_2_O, r.t., 2 h, 30–66%; (ii) acryloyl chloride, K_2_CO_3_, acetone/H_2_O, r.t., 24 h, 41–83%.

The 3,5-bisbenzylidene-4-piperidinones **1a**–**j** were prepared from piperidin-4-one and the corresponding fluorinated aryl aldehydes under basic conditions (Scheme 1). The *N*-acryloyl derivatives **2a**–**j** were generated from **1a**–**j** by treatment with acryloyl chloride in the presence of K_2_CO_3_. All compounds **1** and **2** were obtained as yellow solids.

Compounds **1a**, **1b**, **1g**–**j**, and **2a**–**j** were tested for their antiproliferative activity against human MiaPaCa-2 and Panc-1 pancreatic carcinoma cells (Table 1). Compounds **1c**–**f** showed only low solubility in DMSO and, thus, were unavailable for testing. The 3-fluoro-4-methoxybenzylidene derivative **2c** and the 3,4-difluorobenzylidene analog **2d** showed the highest activities against MiaPaCa-2 and Panc-1 cells, and were more active than the anticancer drug irinotecan. Compounds **2h** and **2i** were also considerably active against the pancreatic carcinoma cells. While in most cases, the activities of the acryloyl derivatives exceeded those of their precursors, **1a** (EF24) was distinctly more active than **2a**, and **1b** exhibited virtually the same activities as **2b**. Additionally, **1j**, **2a**, and **2j** were the least active derivatives. Hence, the trifluoromethyl substituent was superior to the pentafluorosulfanyl substituent regarding antiproliferative activity against pancreatic cancer cells.

**Figure 2:**
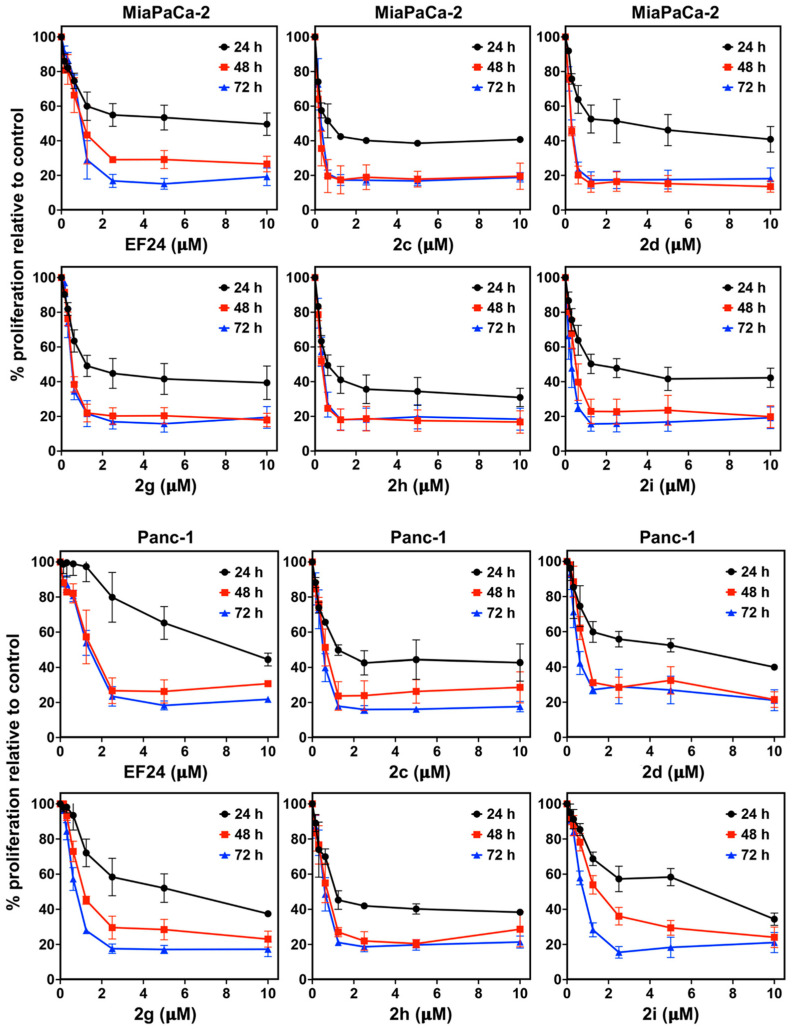
Dose–response curves of EF24 (**1a**), **2c**, **2d**, and **2g**–**i** in MiaPaCa-2 (**top**) and Panc-1 (**bottom**) pancreatic cancer cells at the indicated time points. Each dose–response curve was obtained from three independent experiments.

EF24 (**1a**) and the active *N*-acryloyl compounds **2c**, **2d**, **2g**–**i** were also investigated for their time-dependent activities against MiaPaCa-2 and Panc-1 pancreatic carcinoma cells. Dose–response curves of these compounds after 24 h, 48 h, and 72 h are shown in Figure 2 (for associated *p* values see Table S2). All curcuminoids exerted a high degree of antiproliferative activity after 48 h, which was maintained up to 72 h. After 24 h, compounds **2** showed reduced but still considerable activity. However, EF24 was distinctly less active than the acryl amide derivatives against Panc-1 cells after 24 h. Further, to understand the effects of EF24 and **2d** on noncancerous cells, we used the monocyte cell line (THP1) and immortalized pancreatic ductal cell line (HPNE). EF24 and compound **2d** treatment for 72 h did not induce cytotoxicity to THP1 cells at a concentration of 10 µM (Table S1). Similarly, both curcuminoids did not affect the viability of HPNE cells considerably and inhibited the proliferation of these cells only at doses above 1 µM. The IC_50_ value of **2d** in HPNE cells was ~8 times higher than in cancer cells, while in the case of EF24, it was ~2 times (Table S1). These data suggest that EF24 and compound **2d** were preferentially cytotoxic to pancreatic cancer cells compared to noncancerous cell lines.

**Figure 3:**
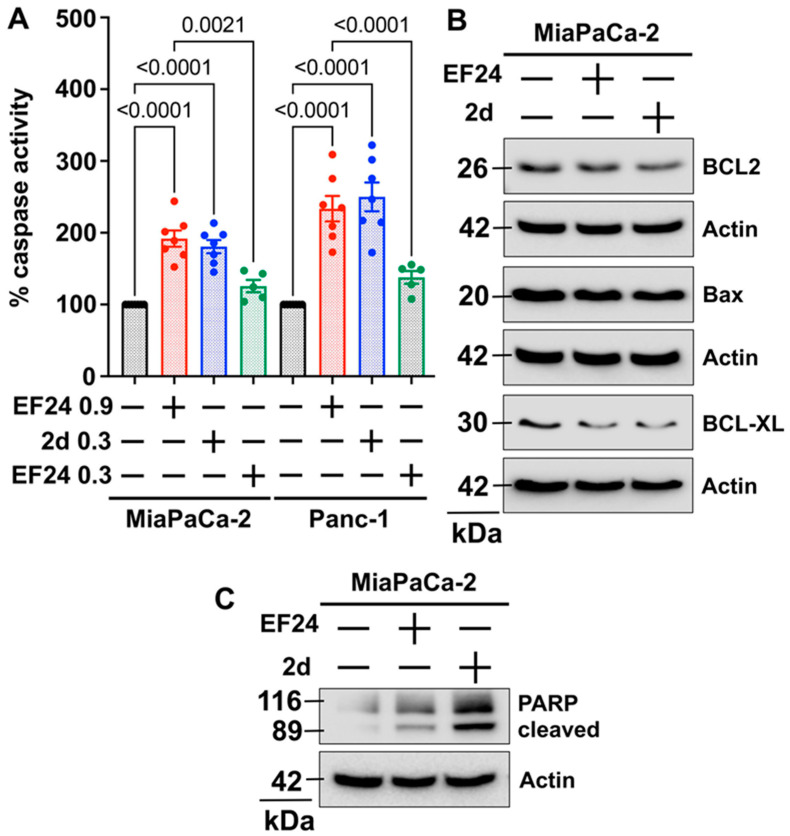
Apoptosis induction by EF24 (**1a**) and **2d** in pancreatic cancer cells. (**A**) Induction of caspase-3 activity by EF24 and **2d** in MiaPaCa-2 and Panc-1 cells (doses in µM). (**B**) Western blot investigation of the expression of apoptosis proteins Bax, BCL2, and BCL-XL in MiaPaCa-2 pancreatic cancer cells upon treatment with EF24 and **2d**. (**C**) Induction of PARP cleavage in MiaPaCa-2 cells upon treatment with EF24 and **2d**.

Apoptosis induction by EF24 and **2d** was studied in MiaPaCa-2 and Panc-1 pancreatic cancer cells (Figure 3). Compound **2d** led to a more robust caspase-3 activation than EF24 at equimolar concentrations of 0.3 µM (Figure 3A). Even three-fold higher doses of EF24 (0.9 µM) only reached (in MiaPaCa-2 cells) or were still less potent (in Panc-1 cells) than **2d**. Both EF24 and **2d** suppressed anti-apoptotic BCL2 and BCL-XL protein expression in MiaPaCa-2 cells, while Bax expression remained unchanged compared with untreated cells (Figure 2B, Figure S1). A distinctly increased poly(ADP-ribose)polymerase (PARP) cleavage in MiaPaCa-2 cells treated with **2d** was observed compared to EF24 and untreated cells (Figure 3C, Figure S1), which aligns with the strong caspase-3 activation by **2d**.

**Figure 4:**
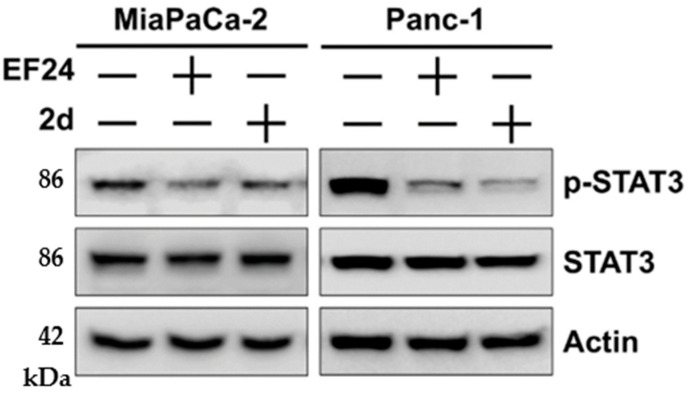
Protein levels of STAT3 and p-STAT3 in MiaPaCa-2 and Panc-1 pancreatic cancer cells upon treatment with EF24 and **2d**.

The influence of EF24 and **2d** on phospho-STAT3 (p-STAT3) levels was investigated in MiaPaCa-2 and Panc-1 pancreatic cancer cells (Figure 4, Figure S2). Both compounds suppressed p-STAT3 levels in these cancer cell lines after 48 h. Compound **2d** appeared to be slightly more active than EF24 in terms of p-STAT3 suppression in Panc-1 cells.

**Figure 5:**
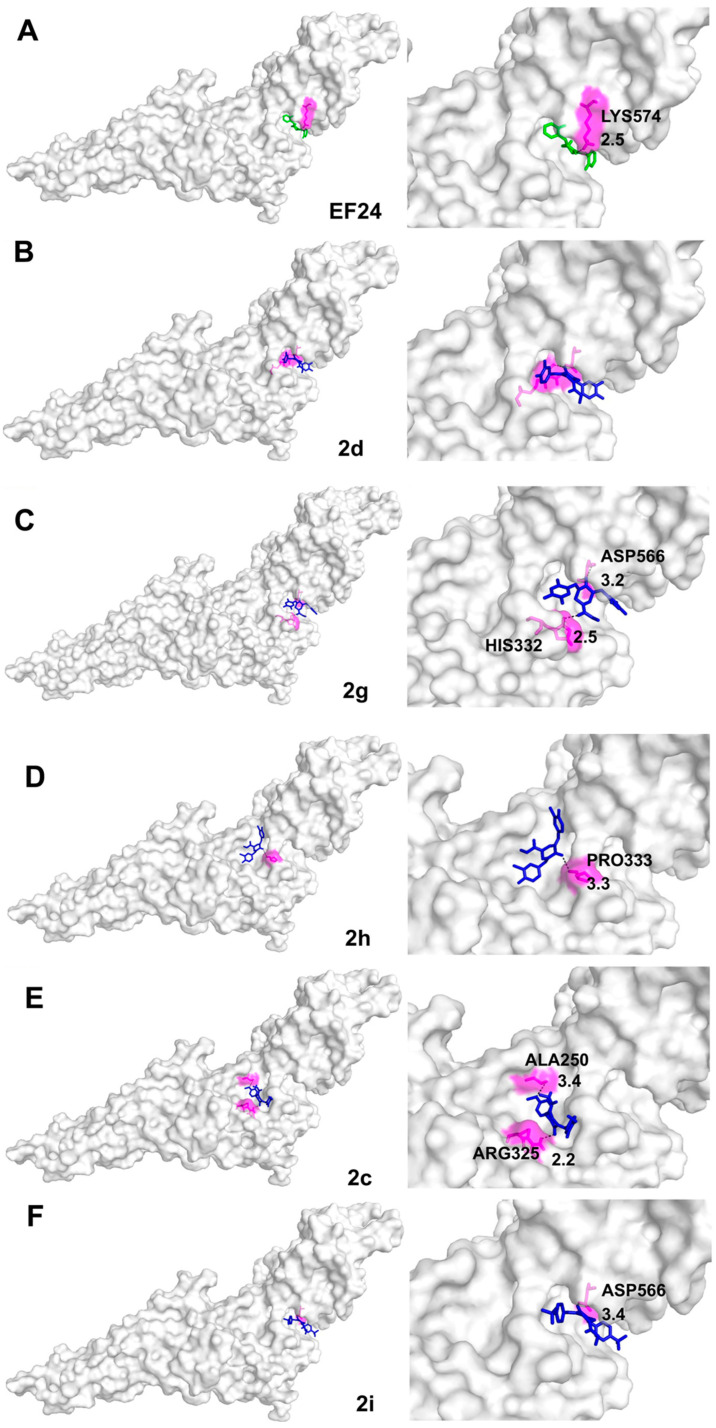
Molecular docking of EF24 (**1a,** (**A**)) and analogs **2c** (**E**), **2d** (**B**), **2g** (**C**), **2h** (**D**), and **2i** (**F**) in STAT3.

Based on the observed effects of EF24 and **2d** on p-STAT3 levels, docking studies of the curcuminoids **1a** (EF24), **2c**, **2d**, and **2g–I** were carried out (Figure 5). EF24 binds to the site of STAT3, which interacts with the SH2-domain of a STAT3 protein. Compounds **2d**, **2g**, and **2i** bind to this site. In contrast, **2c** and **2h** bind differently and occupy a pocket near the EF24 binding site, which belongs to the DNA-binding domain (DBD). The highest binding energy was calculated for **2g** (−8.3 kcal/mol), closely followed by **2d** (−7.9 kcal/mol, Table 2). Furthermore, **1a** (EF24) exhibited a distinctly lower binding energy (−7.3 kcal/mol) when compared with **2g** and **2d**, which form two H bonds with the STAT3 protein in contrast to EF24 with only one H bond. Additionally, **2d**, **2g**, and **2i** interact with ASP566, while EF24 forms an H bond with LYS574.

In terms of the binding site adjacent to the EF24 binding site, it is noteworthy that **2h** showed a higher binding affinity to this site than EF24 to the EF24 binding site. The lowest binding affinity was determined for **2c** (−6.6 kcal/mol), which interacts with the same site as **2h**. However, both compounds differ in the formation and number of H bonds. Compound **2h** forms one H bond with PRO333, while **2c** establishes two H bonds with different amino acids.

## 4. Discussion

The curcuminoids **1a**–**j** and **2a**–**j** were prepared according to straightforward procedures from commercially available starting compounds. Their antiproliferative activities against pancreatic carcinoma cells revealed promising results. Most *N*-acryloyl derivatives **2** were more active than EF24 (**1a**) and the anticancer drug irinotecan. The bis-3-fluoro-4-methoxyphenyl **2c** and the bis-3,4-difluorophenyl derivative **2d** displayed the highest activities with IC_50_ values in the low nanomolar concentration range. Thus, the combination of the *N*-acryloyl-piperidin-4-one with 3,4-difluoro- or 3-fluoro-4-methoxy-substituted phenyl rings appears to be favorable for anti-pancreatic cancer activity, while the 2-fluorophenyl derivative EF24 and its *N*-acryloyl analog **2a** were distinctly less active. In addition, a quicker onset of activity was observed for **2c**, **2d**, **2i**, and other acryl amide derivatives in Panc-1 cells when compared with EF24. Notably, compound **2j** with the pentafluorosulfanyl substituent, also called the ’’super-trifluoromethyl´´ group, was much less active than the trifluoromethyl analog **2i** [46]. This is rather surprising because considerable anticancer effects were recently described for *N*-(m)ethyl-piperidin-4-one-based curcuminoids with SF_5_-substituents [33,34].

Since high antiproliferative activities were observed for compounds **2c**, **2d**, and **2g**–**i**, these active compounds were docked into STAT3, which is a reasonable target of curcumin and structurally related compounds in pancreatic cancer [47,48]. Two different STAT3 binding modes were observed, and **2c** and **2h** interacted with STAT3 differently when compared with the STAT3 binding mode of EF24 and compounds **2d**, **2g**, and **2i**. This discovery has the potential to pave the way for the development of two different groups of anticancer-active curcuminoids depending on their phenyl substitution pattern. In addition, mutant STAT3 proteins might be addressed more efficiently, leading to an improved curcuminoid response in various cancer diseases [49]. EF24 and **2d** also suppressed phospho-STAT3 levels in pancreatic cancer cells, corroborating the docking results obtained for these two curcuminoids. Inhibition of STAT3 was identified as a meaningful strategy to overcome acquired cancer drug resistance [50]. In terms of PDAC, STAT3 inhibition by the quinone-based STAT3 inhibitor napabucasin enhanced the response to chemoradiotherapy [51]. In addition, STAT3 inhibition has the potential to sensitize PDAC to immunotherapy [52]. This is of great relevance since pancreatic cancers are usually weak responders to immune checkpoint inhibitors [53].

Anti-apoptotic mechanisms are a hallmark of cancer, and a considerable induction of apoptosis in pancreatic cancer cells is mandatory for new drug candidates against this cancer disease [54]. Compound **2d** exhibited promising pro-apoptotic activities in pancreatic cancer cells, which distinctly surpassed the apoptosis induction properties of the control compound EF24. Mechanistically, activation of caspase-3, suppression of anti-apoptotic BCL2 and BCL-XL expression, and increased PARP cleavage were detected in pancreatic cancer cells upon treatment with low doses of **2d**. Thus, in terms of apoptosis induction, **2d** adds well to other previously studied apoptosis inducers of curcuminoid-type structures [27,47].

In summary, the activities of **2c**, **2d**, and **2i** against pancreatic carcinoma cells are considerable. Based on these preliminary findings, the described curcuminoids can become treatment options for pancreatic cancer, which is a disease of high mortality where efficient drugs are badly required. Their activities warrant advanced studies in pancreatic carcinoma models, which may include in vivo experiments, as well as testing for DUB inhibitory activity compared with the bis-nitrophenyl analog b-AP15 [36].

## Figures and Tables

**Table 1:** Inhibitory concentrations (IC_50_ in µM) ^1^ of test compounds when applied to pancreatic cancer cells MiaPaCa-2 and Panc-1 after 72 h. Irinotecan served as a positive control. Compounds **1c**–**f** were not tested because of their low solubility.

Compound	MiaPaCa-2	Panc-1
**1a** (EF24)	1.03 ± 0.19	1.52 ± 0.25
**1b**	0.73 ± 0.03	1.13 ± 0.06
**1g**	0.73 ± 0.12	1.32 ± 0.18
**1h**	0.98 ± 0.03	2.03 ± 0.06
**1i**	0.93 ± 0.06	2.78 ± 0.35
**1j**	2.87 ± 0.21	7.32 ± 0.53
**2a**	4.14 ± 0.18	7.37 ± 0.31
**2b**	0.68 ± 0.04	1.05 ± 0.05
**2c**	0.29 ± 0.12	0.51 ± 0.15
**2d**	0.31 ± 0.05	0.53 ± 0.20
**2e**	0.58 ± 0.03	1.02 ± 0.03
**2f**	0.78 ± 0.03	1.13 ± 0.06
**2g**	0.50 ± 0.10	0.74 ± 0.16
**2h**	0.37 ± 0.14	0.64 ± 0.20
**2i**	0.32 ± 0.12	0.77 ± 0.18
**2j**	2.48 ± 0.08	3.78 ± 0.29
**Irinotecan**	1.29 ± 0.36	1.49 ± 0.58

^1^ IC_50_ values ± SD were calculated from the dose–response curves of three independent experiments.

**Table 2:** Binding energies (B.E.) and H bond formation for **1a**, **2c**, **2d**, **2g**–**i** when docked into STAT3.

Compound	B.E. (kcal/mol)	No. of H Bonds	Amino Acids	Distance (Å)
**1a** (EF24)	−7.3	1	LYS574	2.5
**2d**	−7.9	2	ASP566 ARG335	3.2 2.6
**2g**	−8.3	2	HIS332 ASP566	2.5 3.2
**2i**	−7.5	1	ASP566	3.2
**2h**	−7.5	1	PRO333	3.3
**2c**	−6.6	2	ALA250 ARG325	3.4 2.2

## Data Availability

Original data is available from the authors upon request.

## Supplementary Materials

The following supporting information can be downloaded at: https://www.mdpi.com/article/10.3390/pharmaceutics15071921/s1, Tables S1 and S2, Figures S1 and S2, original NMR and mass spectra of the new compounds. Table S1. Inhibitory concentrations (IC_50_ in µM)^1^ of EF24 and compound **2d** when applied to immortalized normal pancreatic ductal cells HPNE and monocyte cell line THP-1 after 72 h. Table S2. Statistical analysis of dose-response curves of EF24 (**1a**), **2c**, **2d**, and **2g**–**i** in MiaPaCa-2 and Panc-1 pancreatic cancer cells at the indicated time points. Figure S1. Apoptosis induction by EF24 (**1a**) and **2d** in pancreatic cancer cell line MiaPaCa-2. Western blots of apoptotic proteins were quantified using Bio-Rad’s Image lab software and expressed as compared to actin in arbitrary units. Figure S2. Protein levels of STAT3 and p-STAT3 in MiaPaCa-2 and Panc-1 pancreatic cancer cells upon treatment with EF24 and **2d**. Western blot of p-STAT3 protein was quantified using Bio-Rad’s Image lab software and expressed as compared to total STAT3 in arbitrary units.
