# Supplementary material for: Fluorinated and N-Acryloyl-Modified 3,5-Di[(E)-benzylidene]piperidin-4-one Curcuminoids for the Treatment of Pancreatic Carcinoma
Source: Pharmaceutics. 2023 Jul 11;15(7):1921. doi: 10.3390/pharmaceutics15071921 (PMC10385166; doi:10.3390/pharmaceutics15071921)

Supplementary Material

Fluorinated and *N*-Acryloyl-Modified 3,5-Di[(*E*)-benzylidene]piperidin-4-one curcuminoids for the treatment of pancreatic carcinoma

Hindole Ghosh [1], Sangita Bhattacharyya [1], Rainer Schobert [2], Prasad Dandawate [1] and Bernhard Biersack [2,*]

[1]Cancer Biology, University of Kansas Medical Center, 3901 Rainbow Boulevard, Kansas City, KS 66160, USA; hghosh@kumc.edu, sbhattacharyya@kumc.edu, pdandawate@kumc.edu
[2]Organic Chemistry 1, University of Bayreuth, Universitätsstrasse 30, 95440 Bayreuth, Germany; rainer.schobert@uni-bayreuth.de, bernhard.biersack@uni-bayreuth.de
*Correspondence: bernhard.biersack@uni-bayreuth.de, bernhard.biersack@yahoo.com

Table S1. Inhibitory concentrations (IC_{50} in μM)[1] of EF24 and compound **2d** when applied to immortalized normal pancreatic ductal cells HPNE and monocyte cell line THP-1 after 72 h.

Cell lines	EF24	2d
**HPNE**	2.3 ± 0.64	2.05 ± 0.39
**THP1**	> 10 μM	>10 μM

[1] IC_{50} values ± SD were calculated from the dose-response curves of three independent experiments.

Table S2. Statistical analysis of dose-response curves of EF24 (**1a**), **2c**, **2d**, and **2g–i** in MiaPaCa-2 and Panc-1 pancreatic cancer cells at the indicated time points.

**p values**							
		**MiaPaCa-2**			**Panc-1**		
**EF24**	Concentration (µM)	**24 h**	**48 h**	**72 h**	**24 h**	**48 h**	**72 h**
	0						
	0.15625	0.02802	0.000758	0.068558	0.825069	0.003479	0.02982
	0.3125	0.001909	0.160187	0.033967	0.965481	0.000306	0.0442
	0.625	0.00463	0.078627	0.0003	0.879541	0.030057	0.00482
	1.25	0.008242	0.077355	0.003001	0.772516	0.048285	0.005496
	2.5	0.002328	0.000443	2.45E-05	0.22838	0.00056	0.000443
	5	0.002841	0.005327	1.07E-05	0.02047	0.000374	1.51E-05
**2c**	0						
	0.15625	0.001165	0.015913	0.067368	0.054244	0.063715	0.29907
	0.3125	0.010269	0.02312	0.00322	0.013919	0.023115	0.184221
	0.625	0.038427	0.013691	0.000206	0.001012	0.04302	0.040358
	1.25	0.000617	0.009385	0.00011	0.003523	0.011118	7.86E-05
	2.5	0.000689	0.007545	0.000111	0.013841	0.011989	0.001139
	5	0.000746	0.003014	7.61E-05	0.037907	0.008141	0.00069
**2d**	0						
	0.15625	0.070948	0.016849	0.026471	0.609201	0.009397	0.390053
	0.3125	0.022205	0.020083	0.000238	0.459126	0.052244	0.095258
	0.625	0.066718	0.015383	0.014833	0.093038	0.011609	0.004116
	1.25	0.040853	0.011428	0.00848	0.002466	8.68E-05	2.41E-09
	2.5	0.087131	0.015555	0.009426	0.000557	3.34E-05	1.62E-05
	5	0.039226	0.011247	0.011479	0.000214	2.79E-05	1.59E-05
**2g**	0						
	0.15625	9.21E-05	0.010536	0.722117	0.431106	0.203007	0.200738
	0.3125	0.007664	0.003592	0.033492	0.59158	0.192595	0.073197
	0.625	0.004725	0.000152	0.000186	0.477306	0.012689	0.008118
	1.25	0.001152	0.000109	0.000459	0.023684	3.61E-05	4.7E-06
	2.5	0.00304	7.17E-05	3.84E-05	0.017397	0.000892	1.76E-05
	5	0.002798	7.05E-05	6.28E-05	0.004106	0.000532	8.85E-06
**2h**	0						
	0.15625	2.99E-05	0.024734	0.074793	0.152711	0.246178	0.112263
	0.3125	0.00783	0.024678	0.007062	0.237733	0.164897	0.037765
	0.625	0.013537	0.004521	0.050885	0.021351	0.056353	0.005605
	1.25	0.016891	0.013405	0.019606	0.00918	0.001286	2.08E-11
	2.5	0.015843	0.016675	0.021269	0.001142	0.004546	8.57E-06
	5	0.014552	0.013094	0.02628	0.002354	0.000816	1.15E-05
**2i**	0						
	0.15625	0.054276	0.006122	0.067449	0.36447	0.20061	0.008287
	0.3125	0.019464	0.218863	0.00903	0.19091	0.241849	0.002689
	0.625	0.013611	0.018439	0.114977	0.012352	0.024517	0.000628
	1.25	0.000869	0.00129	0.052726	0.001207	1.56E-06	1.35E-05
	2.5	0.000674	0.001366	0.057217	0.004101	8.69E-05	1.4E-06
	5	0.000929	0.00277	0.064769	0.00103	0.000294	0.000231

Figure S1. Apoptosis induction by EF24 (**1a**) and **2d** in pancreatic cancer cell line MiaPaCa-2. Western blots of apoptotic proteins were quantified using Bio-Rad's Image lab software and expressed as compared to actin in arbitrary units.

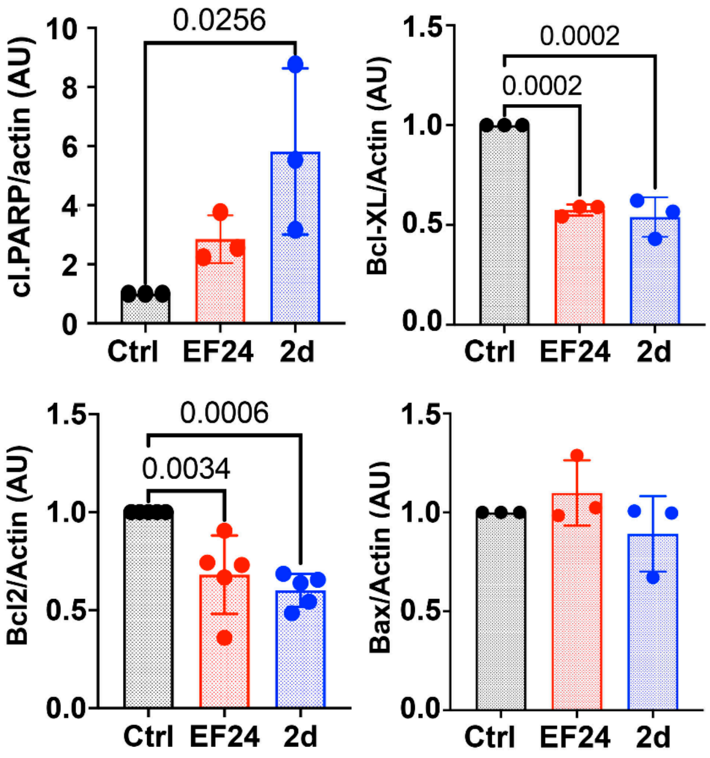

Figure S2. Protein levels of STAT3 and p-STAT3 in MiaPaCa-2 and Panc-1 pancreatic cancer cells upon treatment with EF24 and **2d**. Western blot of p-STAT3 protein was quantified using Bio-Rad's Image lab software and expressed as compared to total STAT3 in arbitrary units.

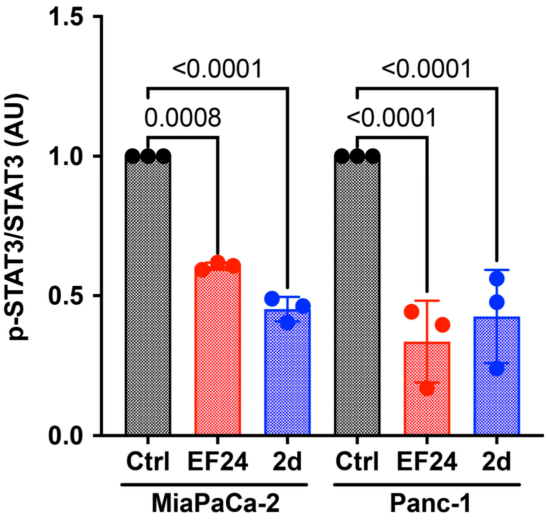

^{1}H NMR spectrum of **1c**

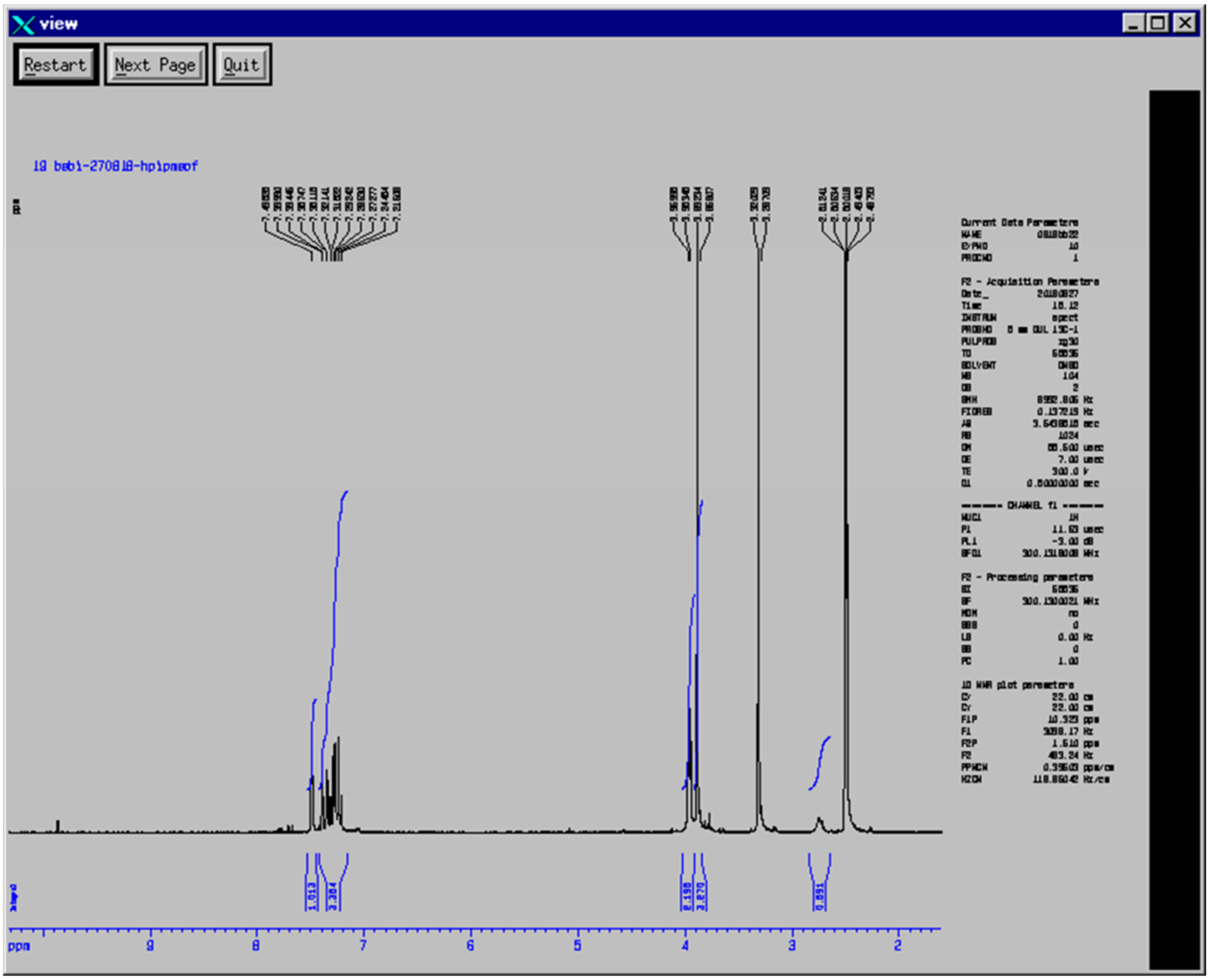

^{13}C NMR spectrum of **1c**

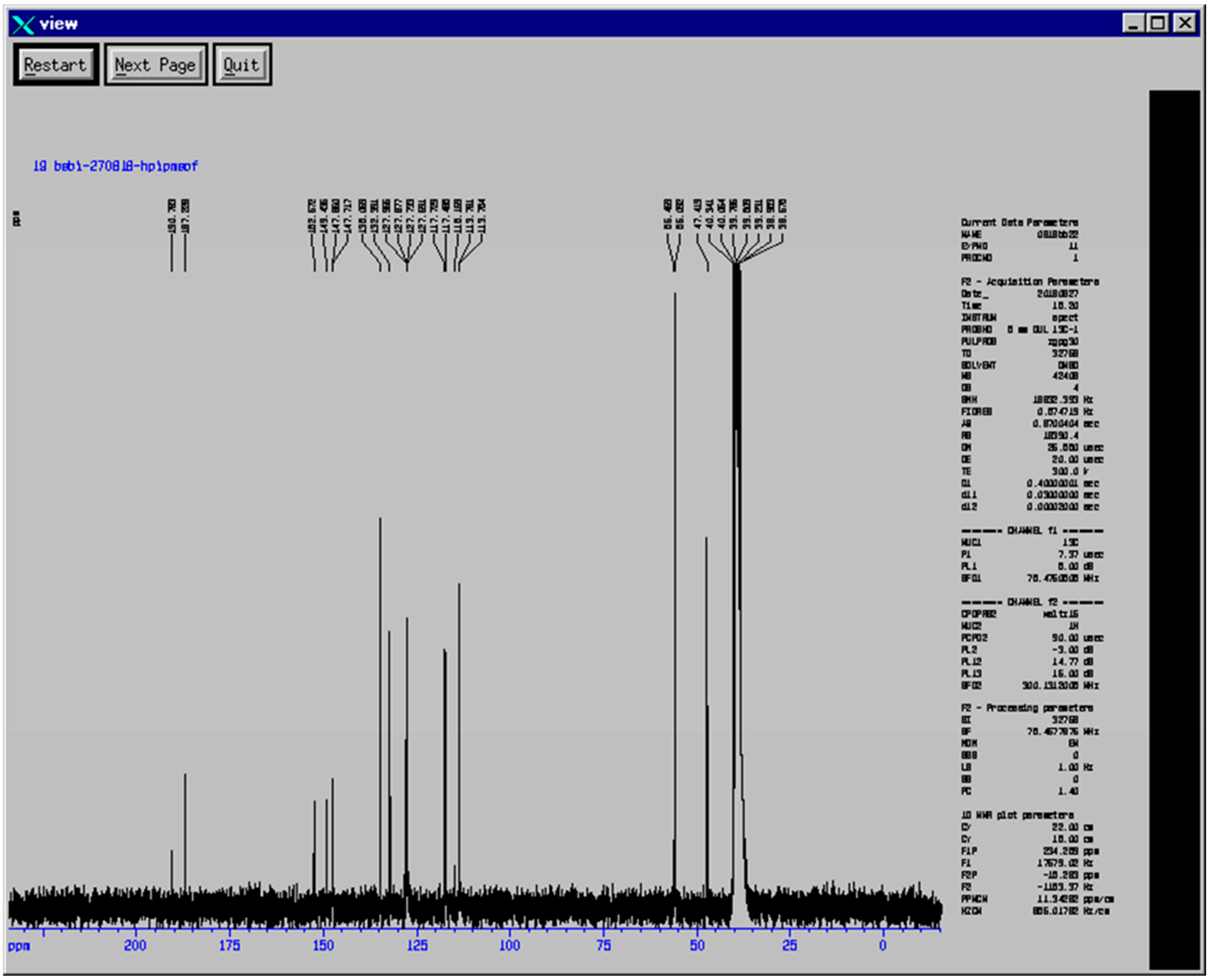

^{1}H NMR spectrum of **1e**

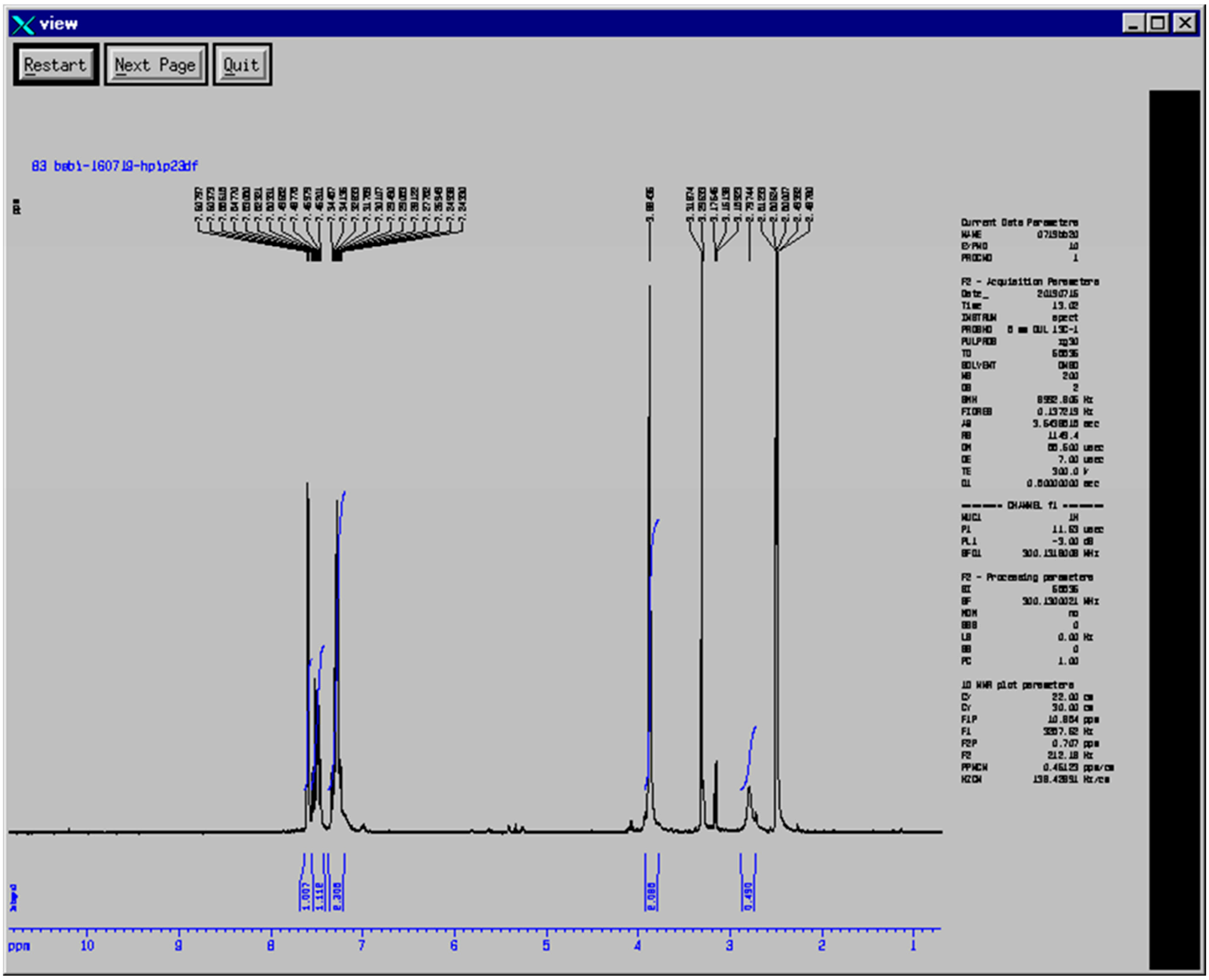

^{13}C NMR spectrum of **1e**

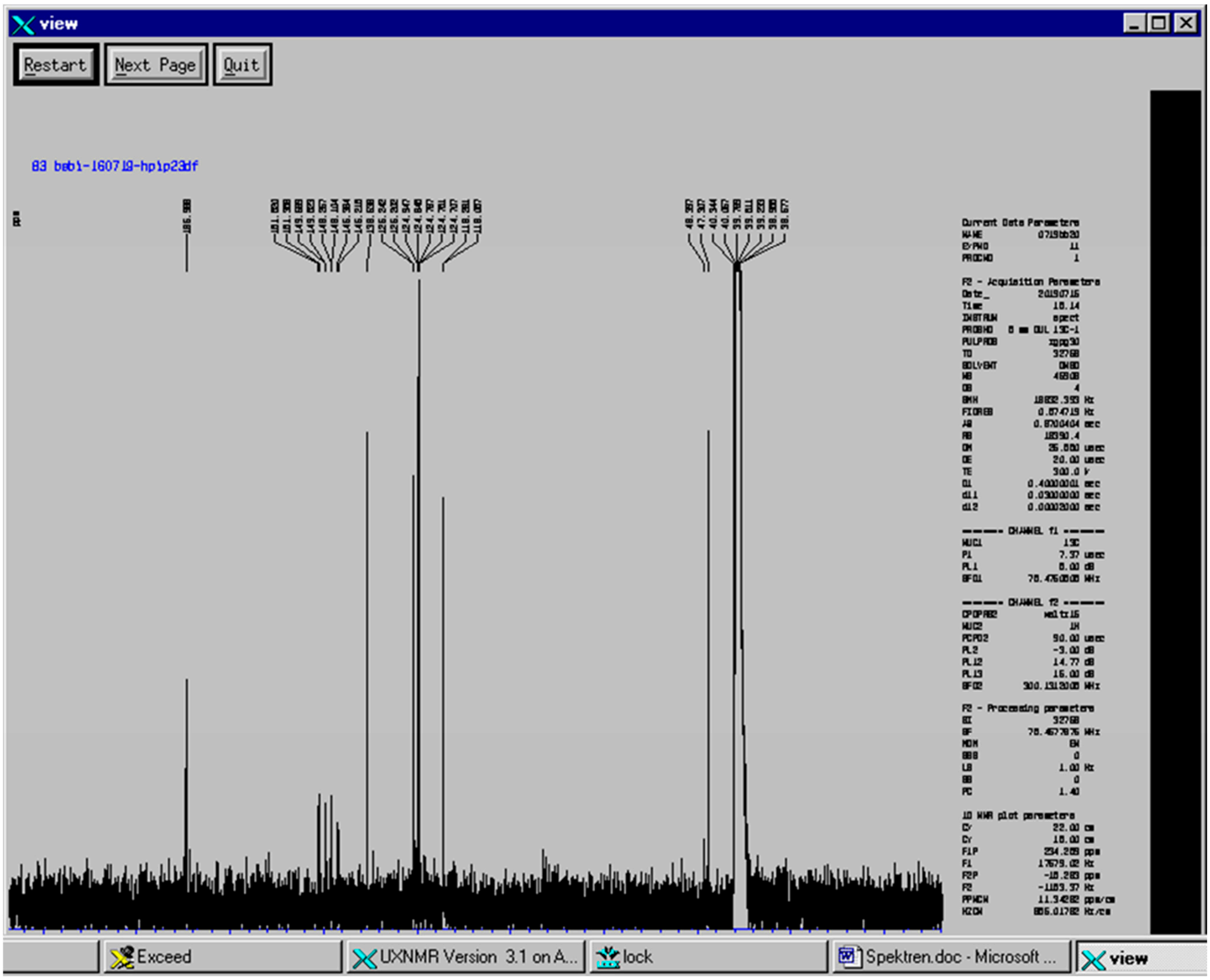

^{1}H NMR spectrum of **1f**

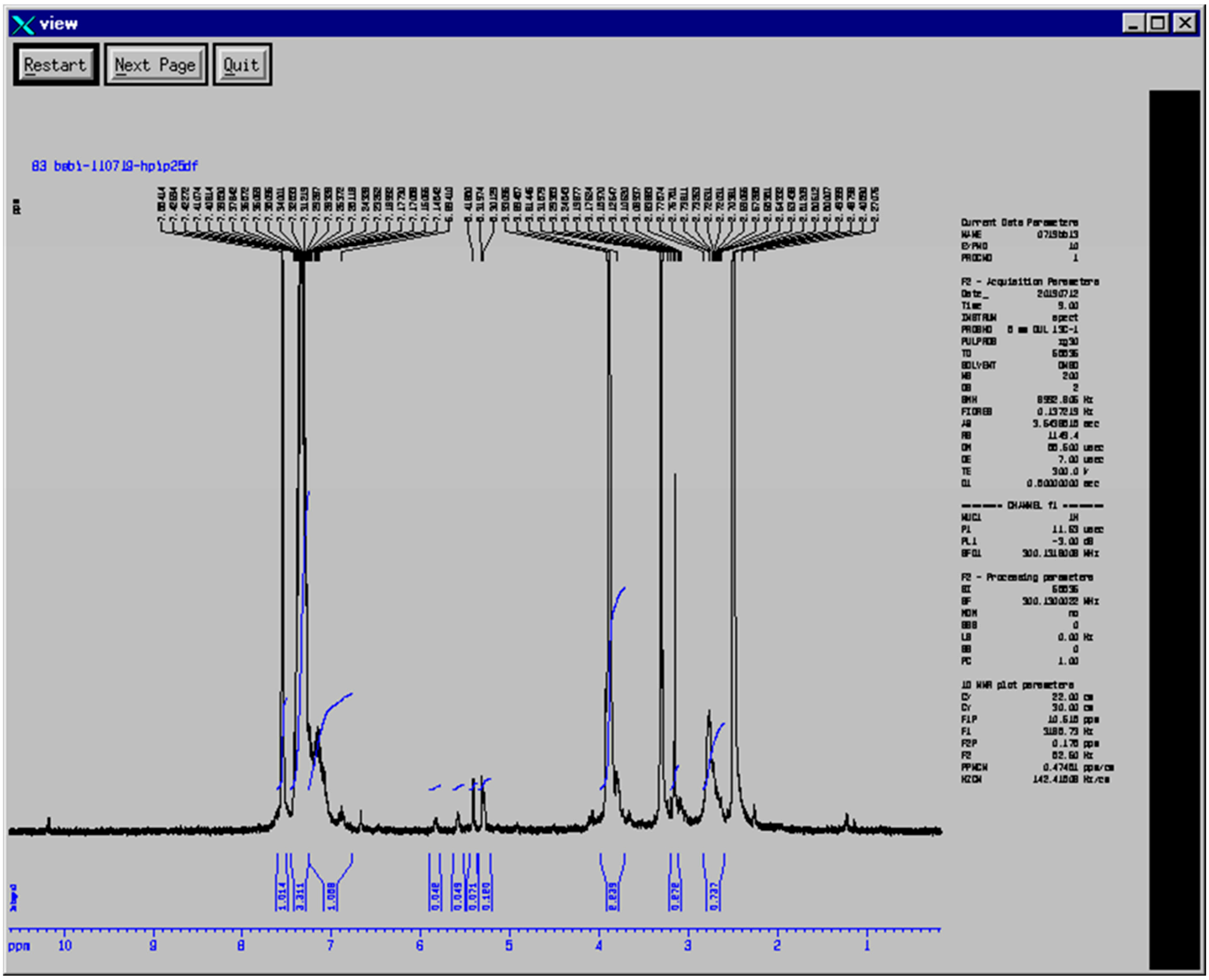

^{13}C NMR spectrum of **1f**

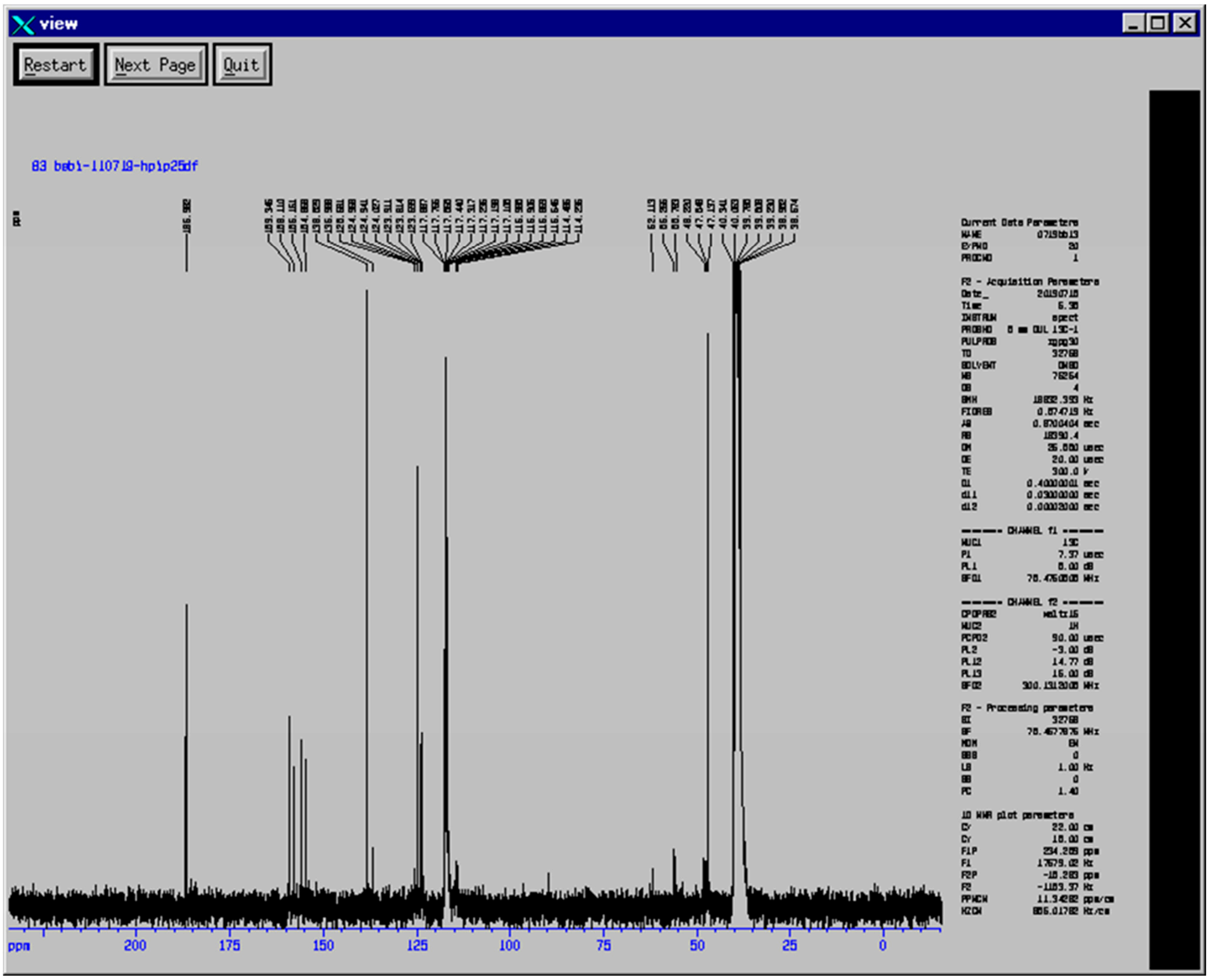

^{1}H NMR spectrum of **1g**

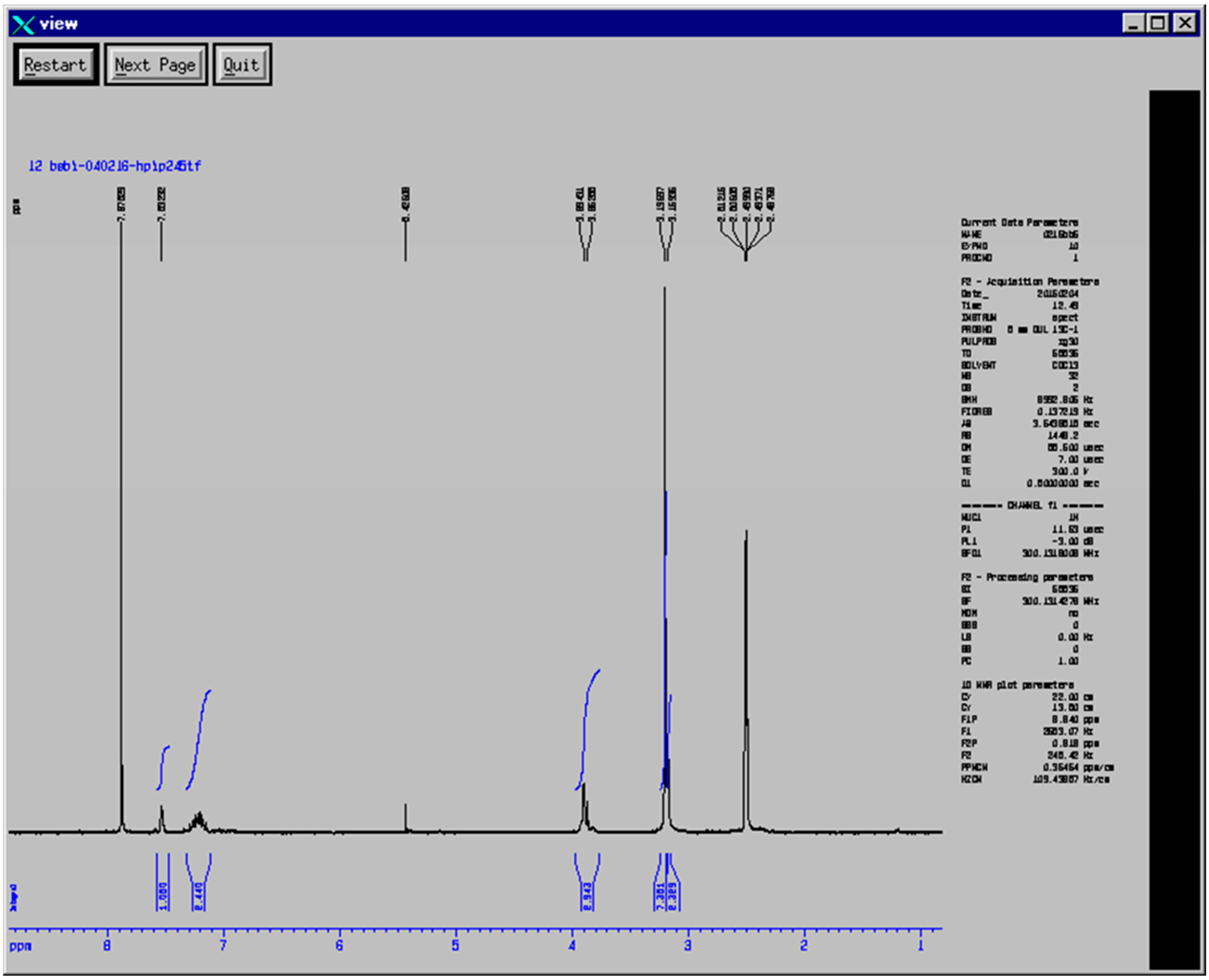

^{13}C NMR spectrum of **1g**

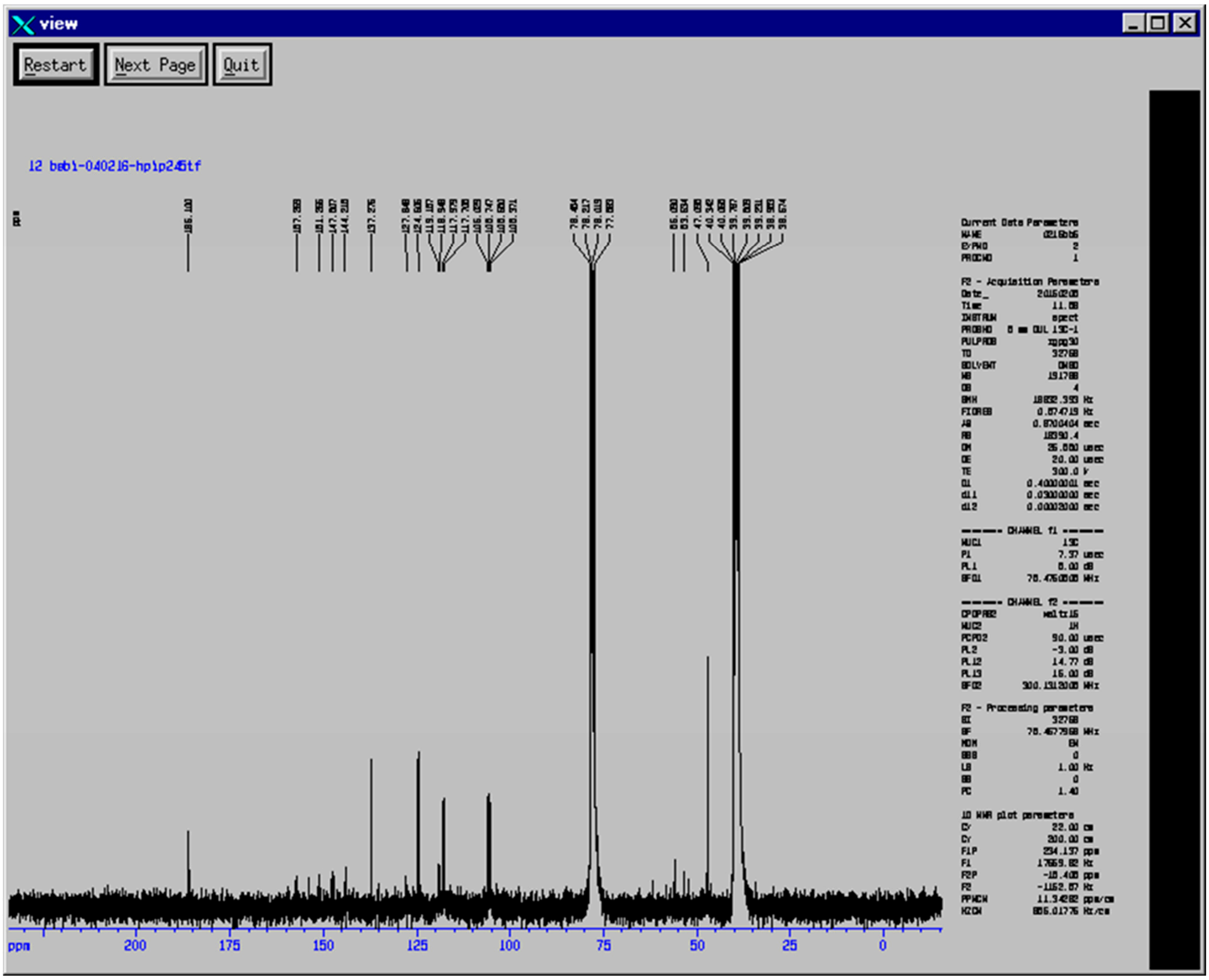

^{1}H NMR spectrum of **1h**

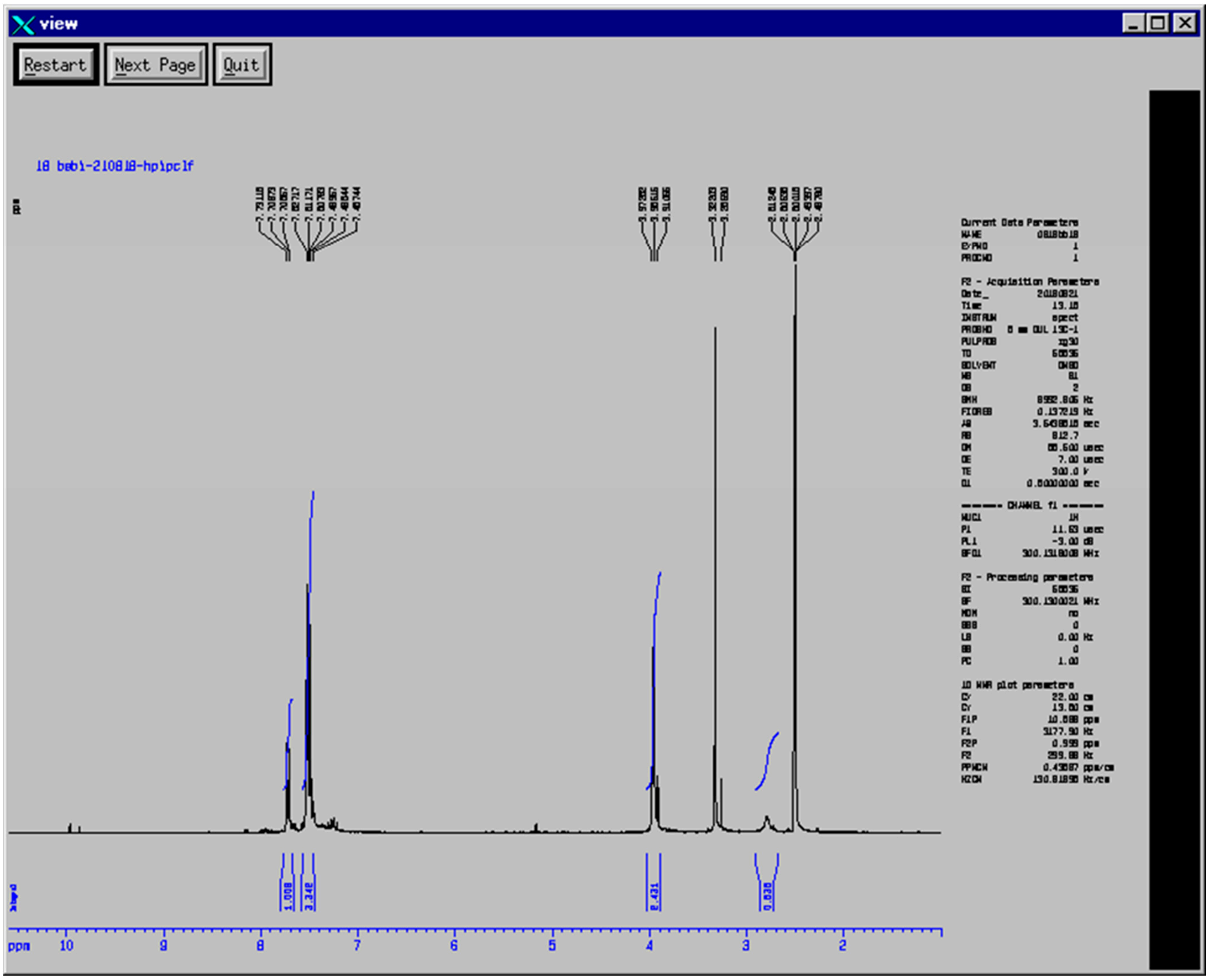

^{13}C NMR spectrum of **1h**

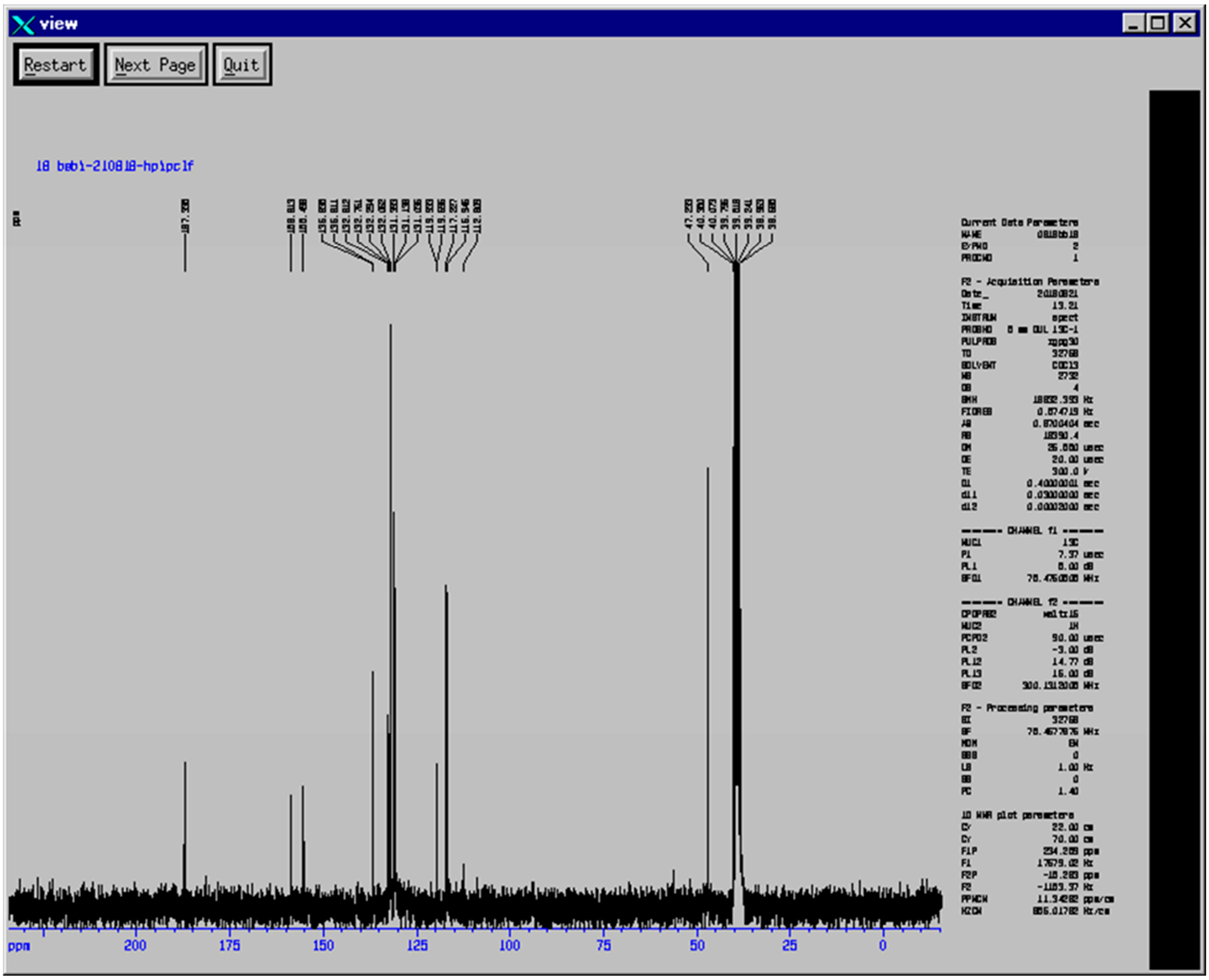

^{1}H NMR spectrum of **2b**

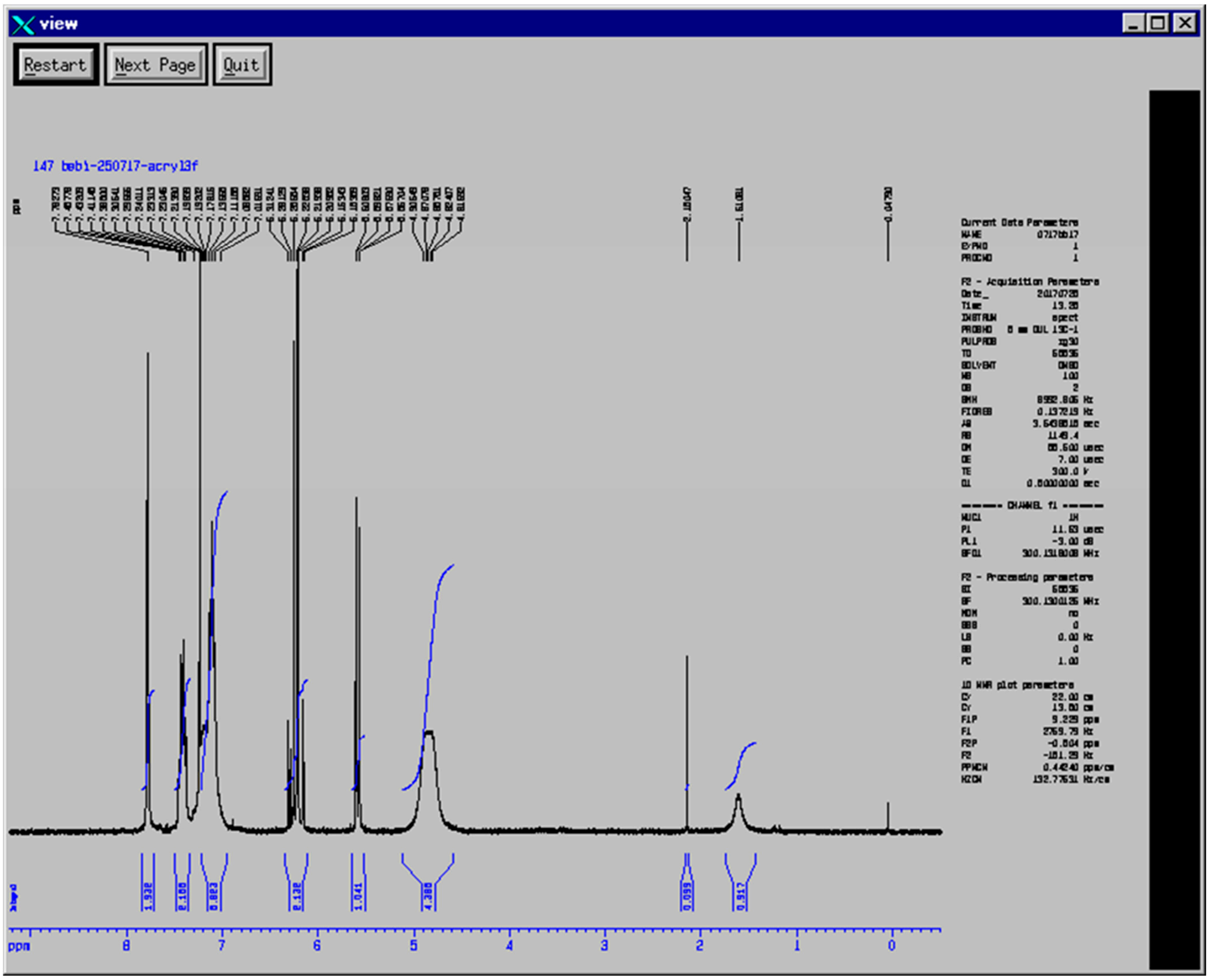

^{13}C NMR spectrum of **2b**

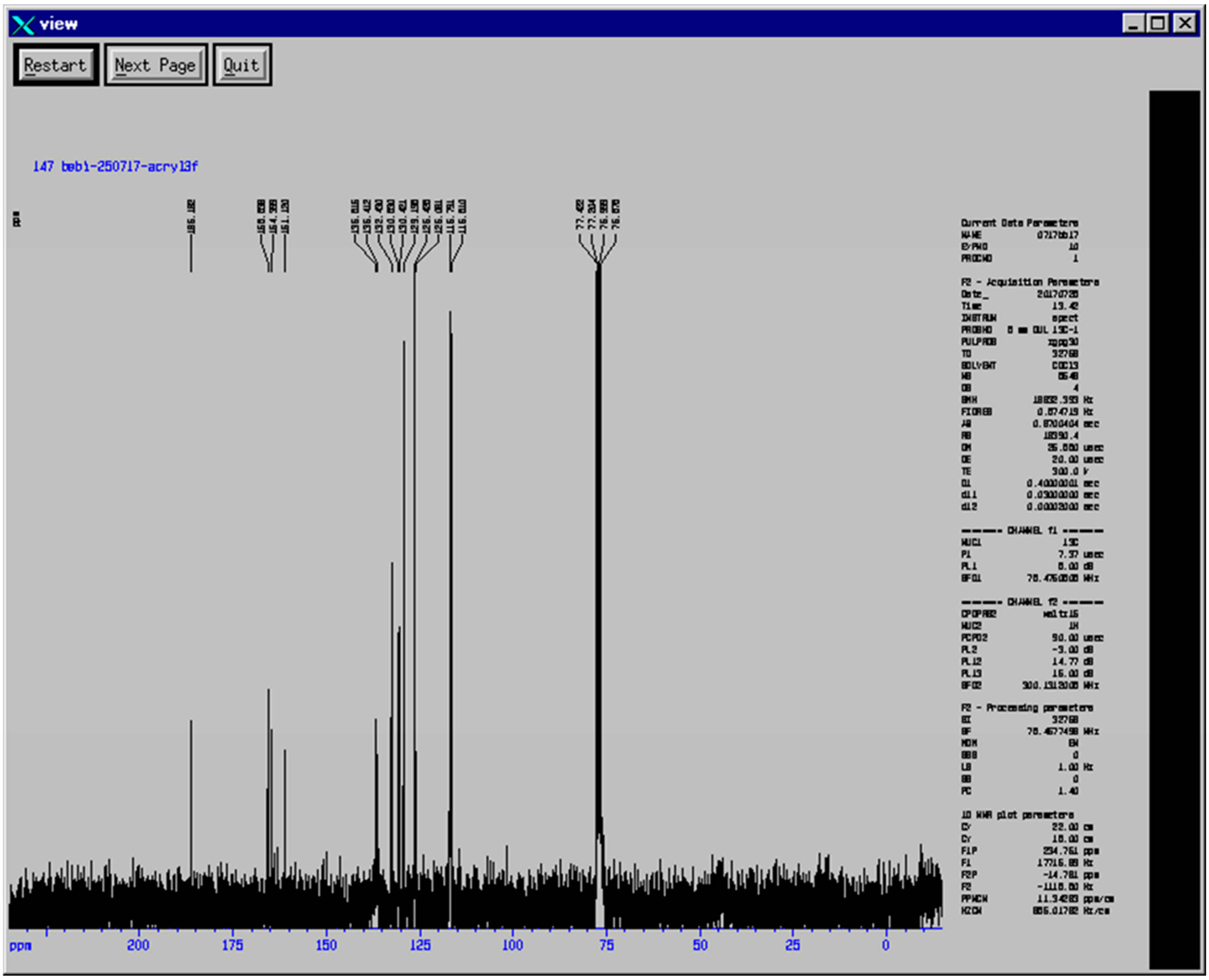

^{1}H NMR spectrum of **2c**

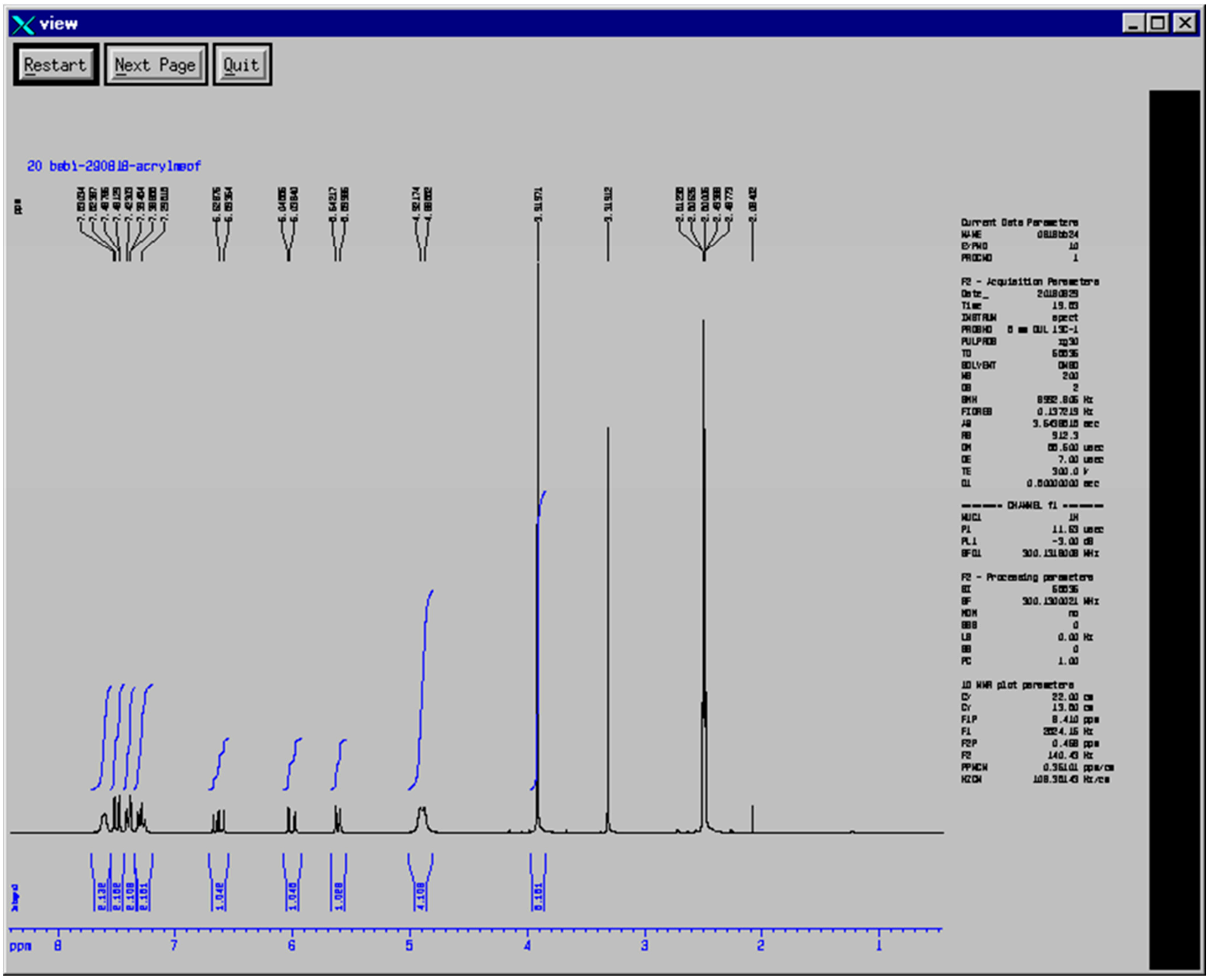

^{13}C NMR spectrum of **2c**

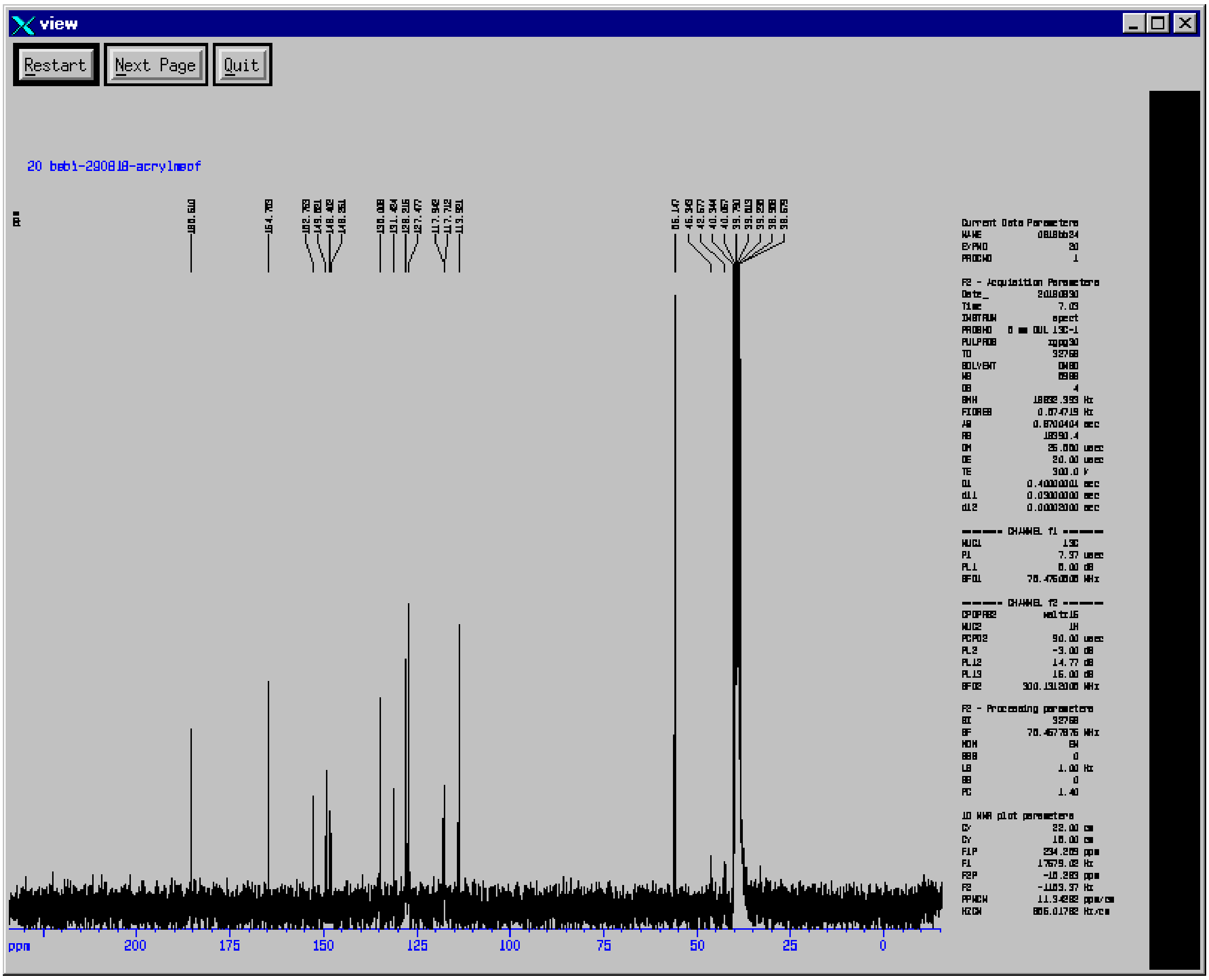

^{1}H NMR spectrum of **2e**

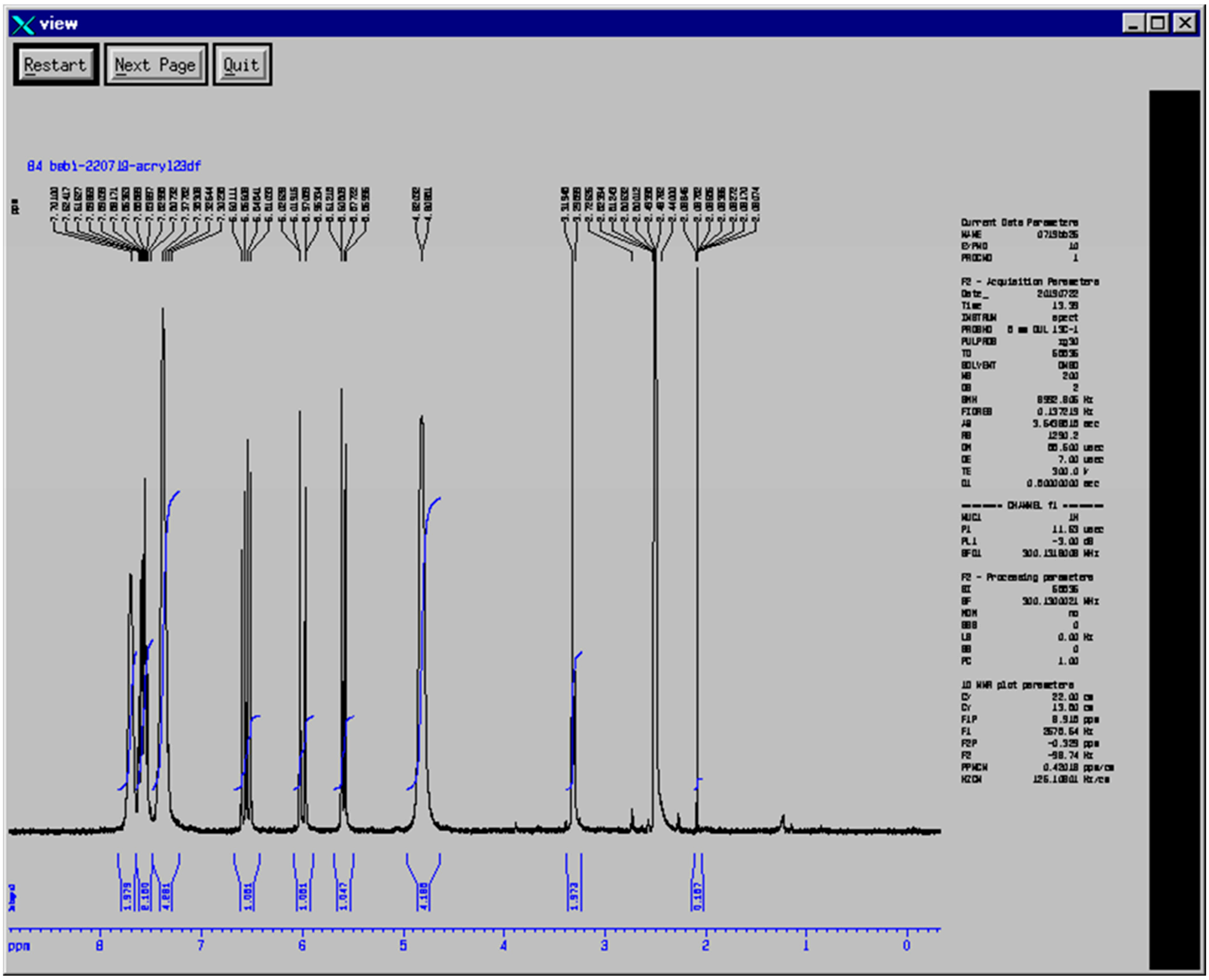

^{13}C NMR spectrum of **2e**

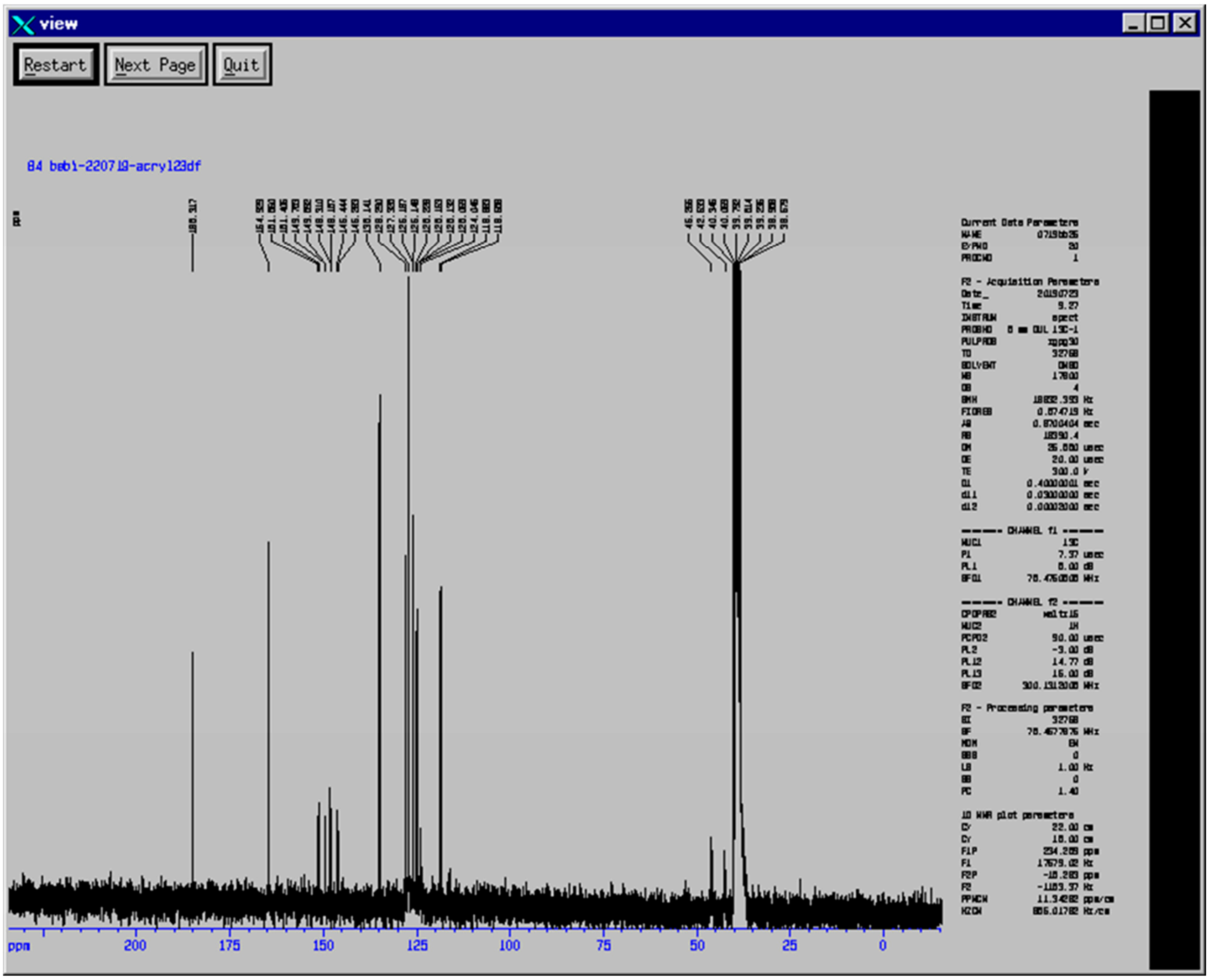

^{1}H NMR spectrum of **2f**

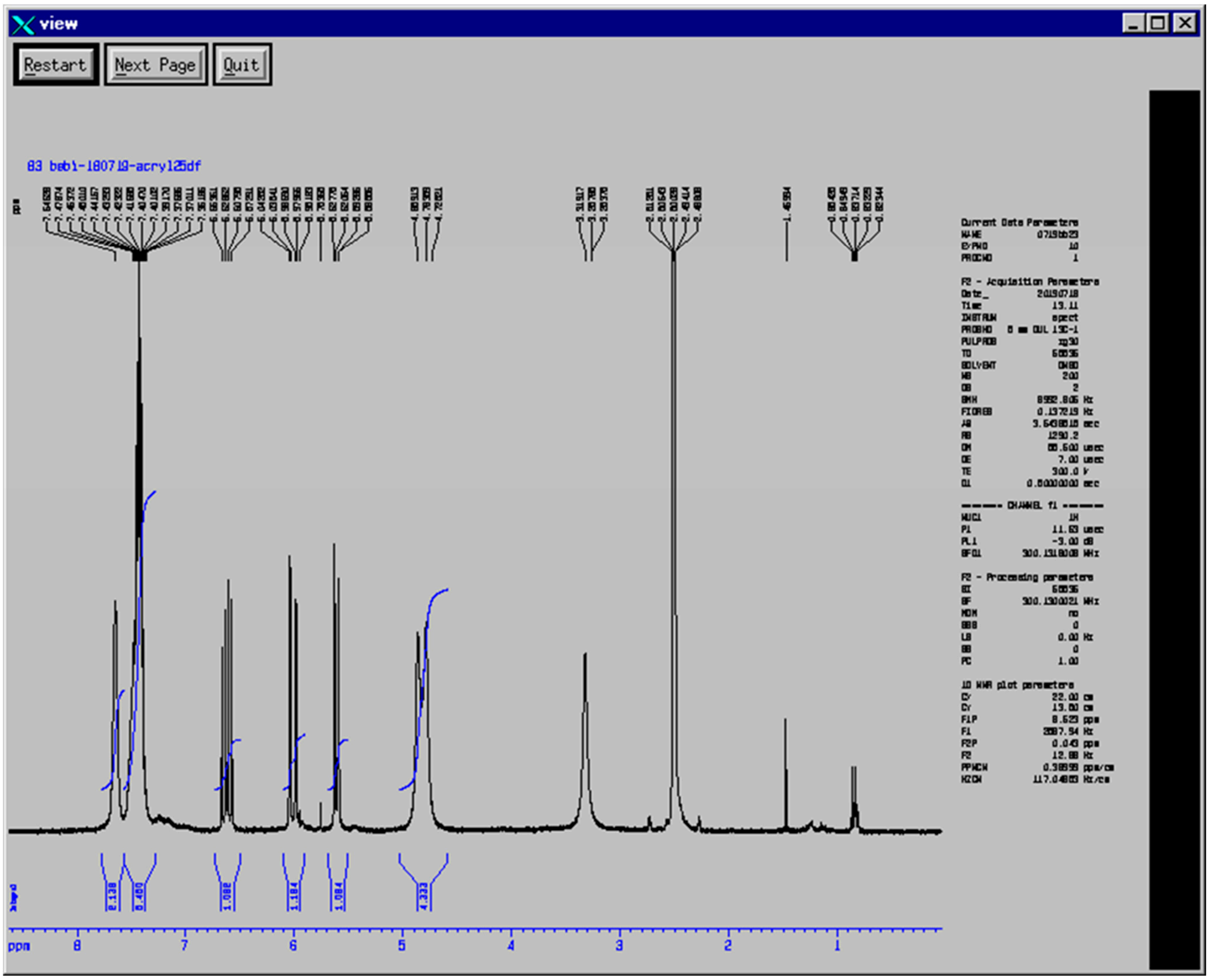

^{13}C NMR spectrum of **2f**

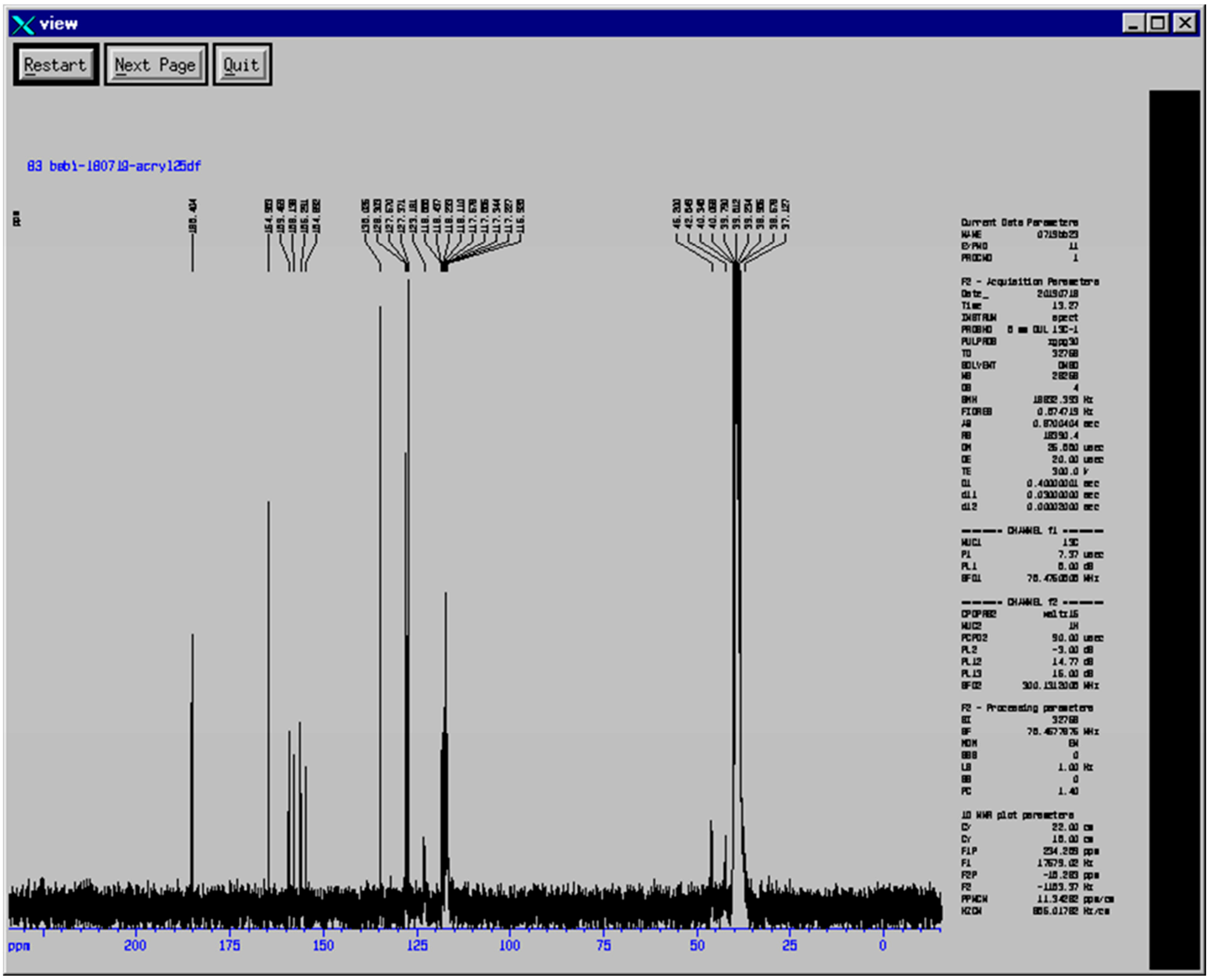

^{1}H NMR spectrum of **2g**

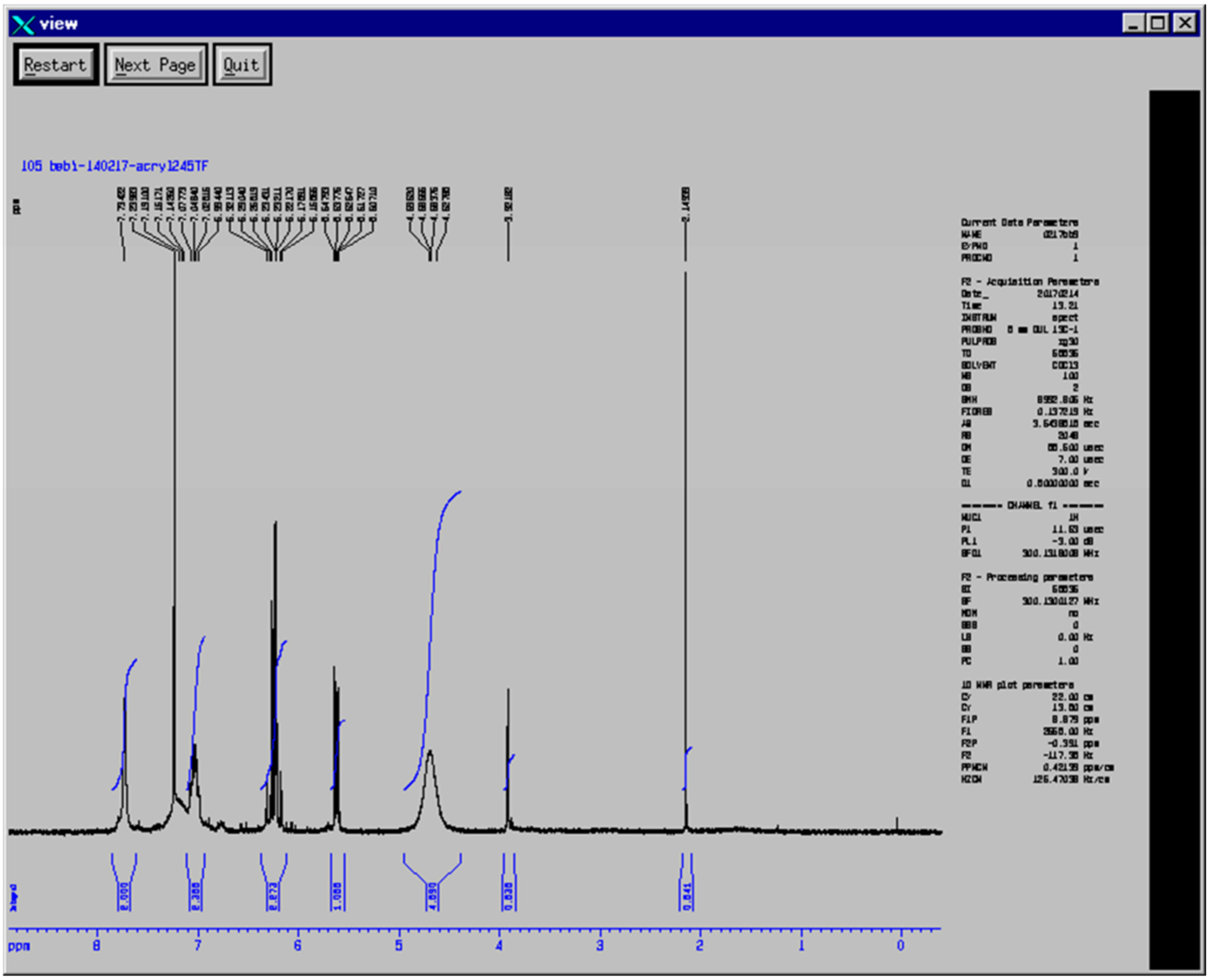

^{13}C NMR spectrum of **2g**

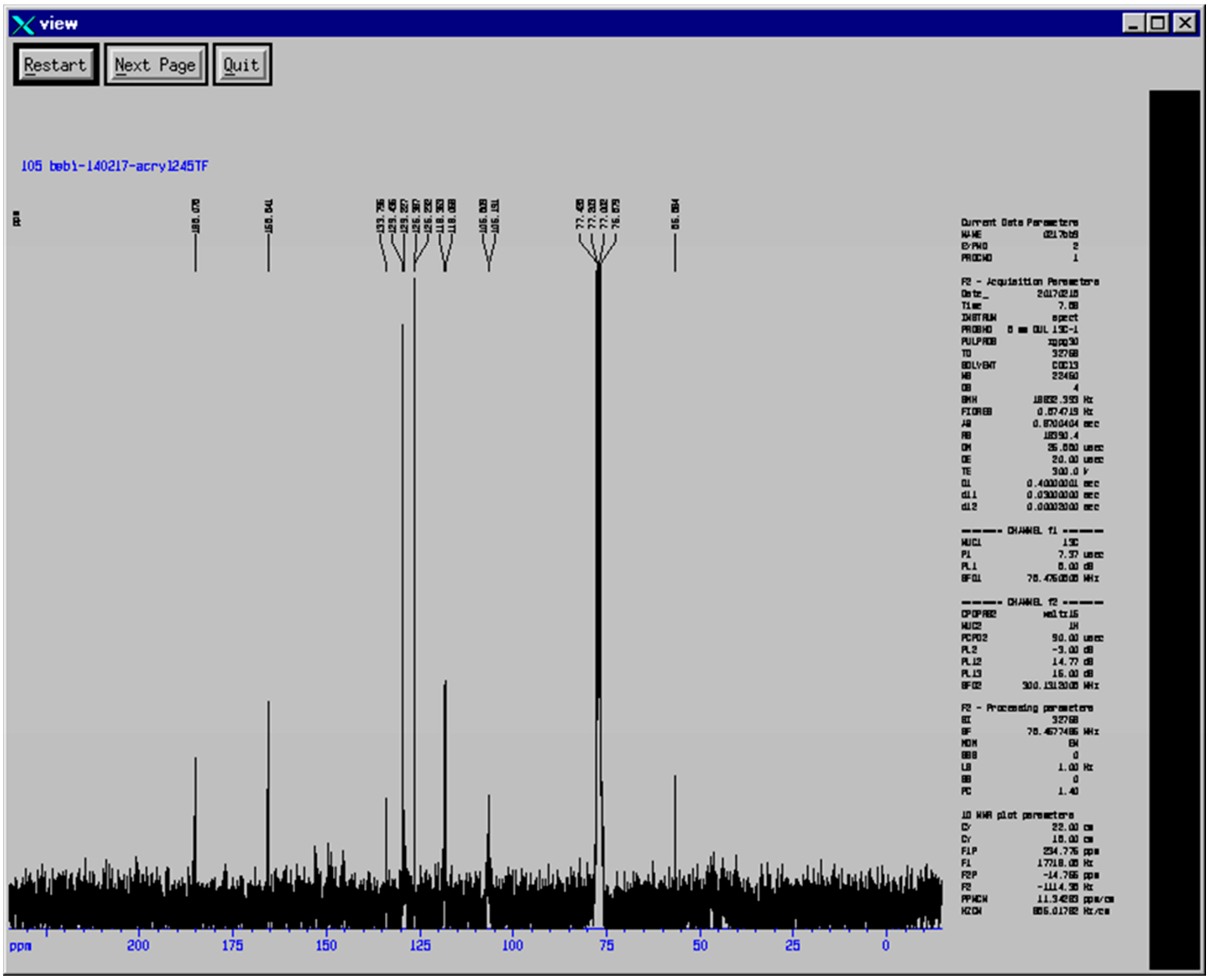

^{1}H NMR spectrum of **2h**

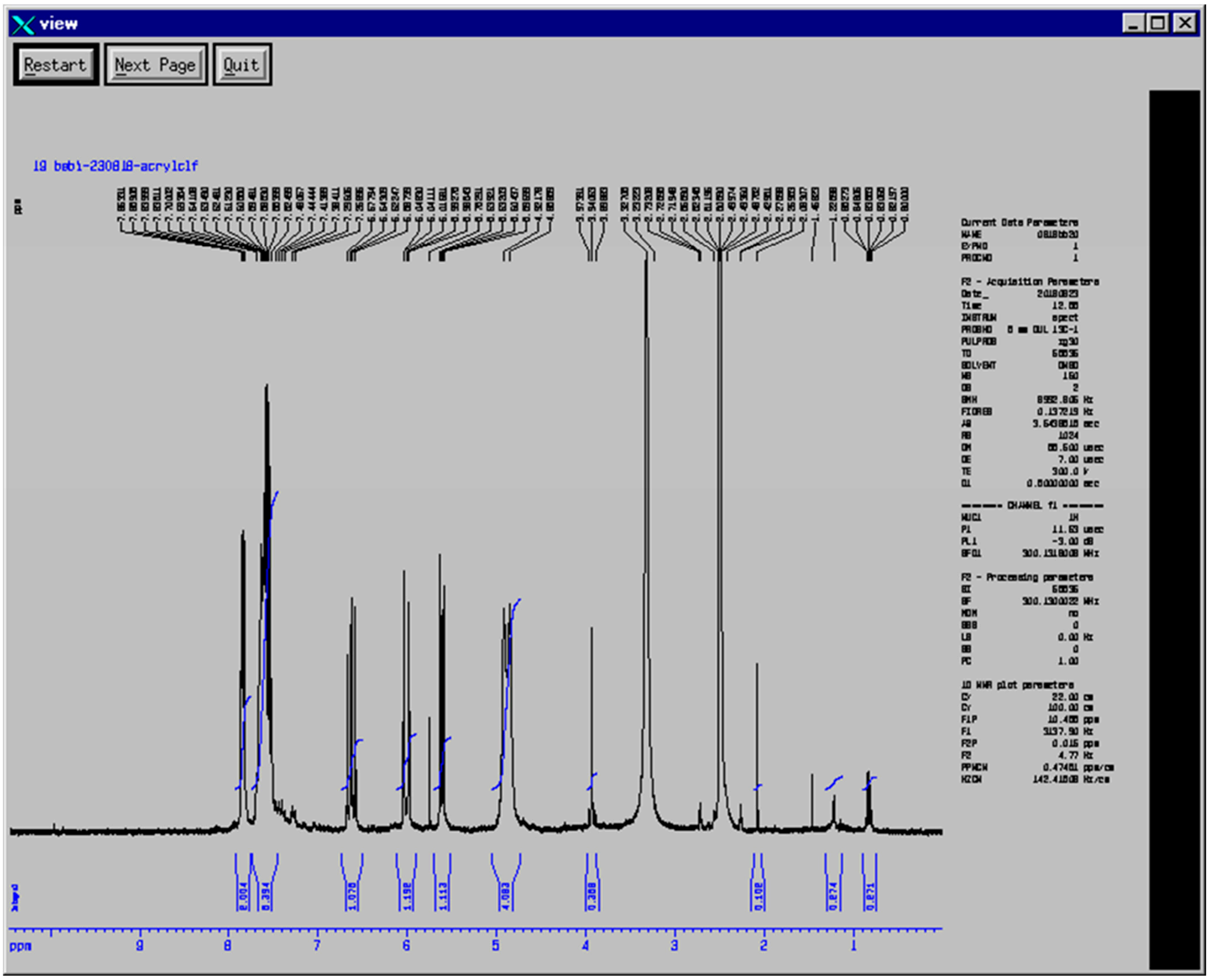

^{13}C NMR spectrum of **2h**

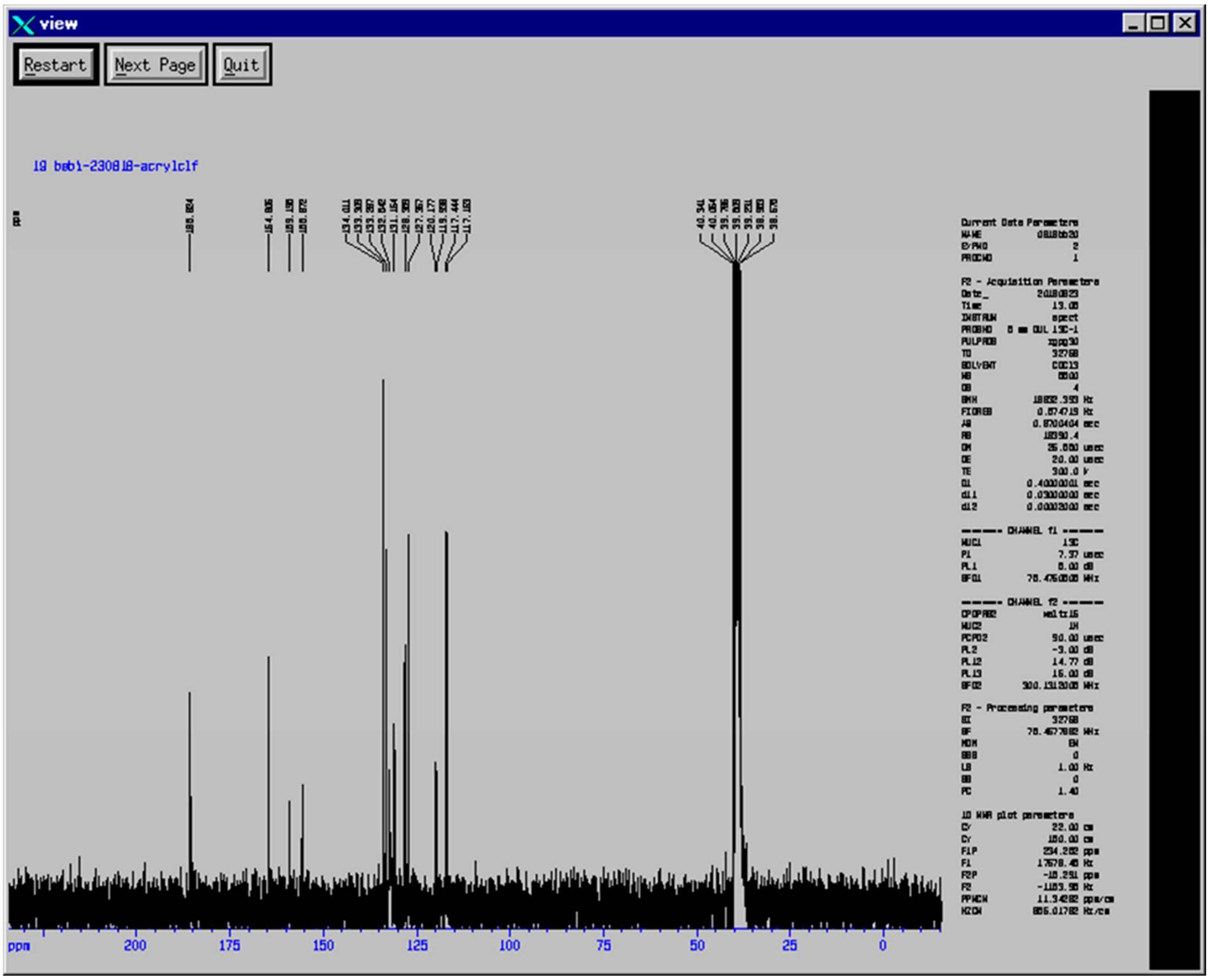

ESI-MS spectrum of **2b**

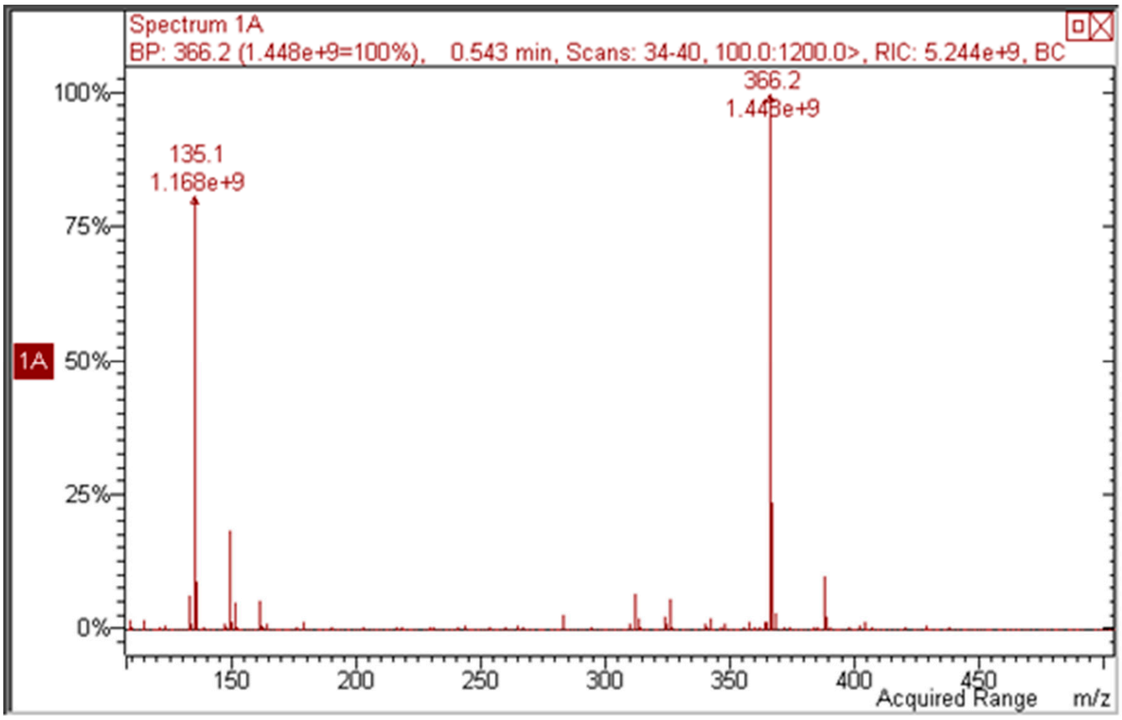

EI mass spectrum of **1c**

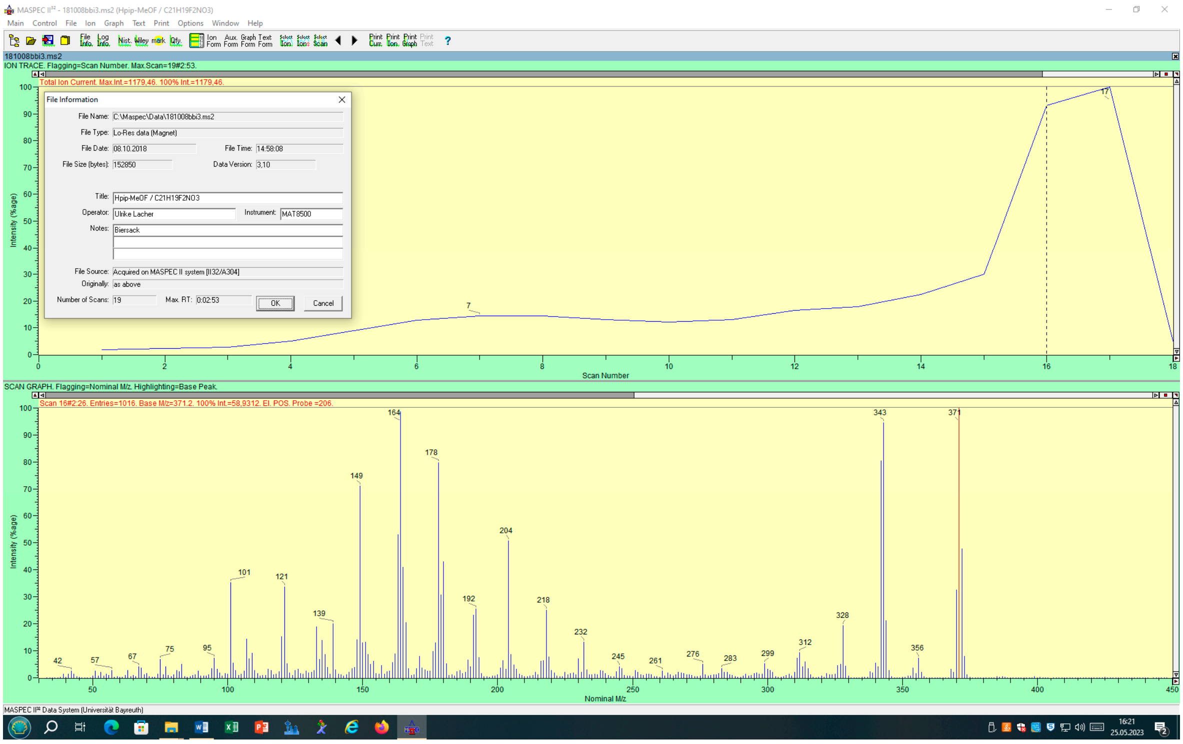

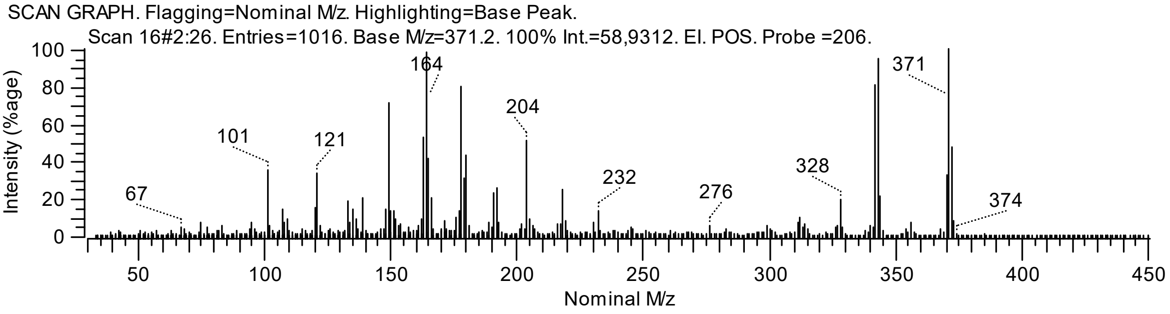

EI mass spectrum of **1e**

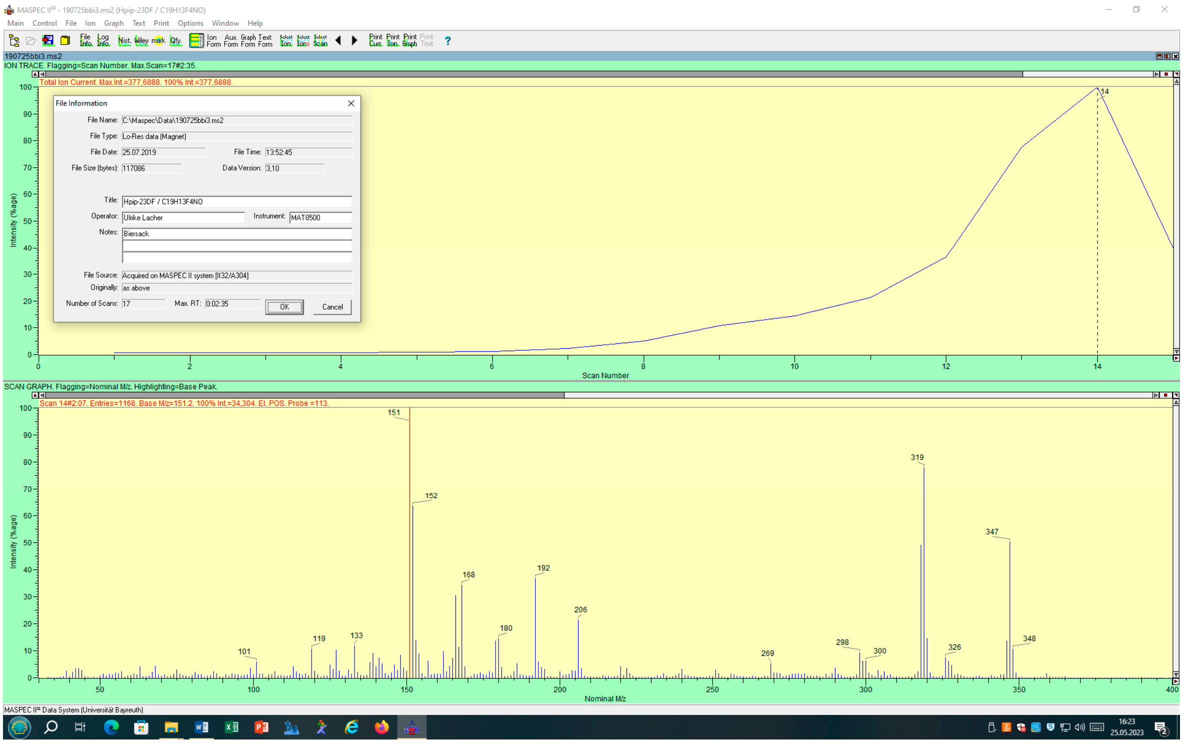

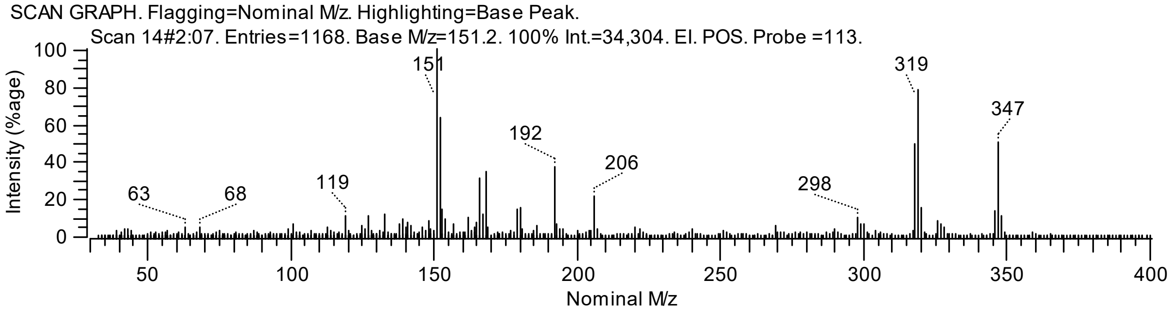

EI mass spectrum of **1f**

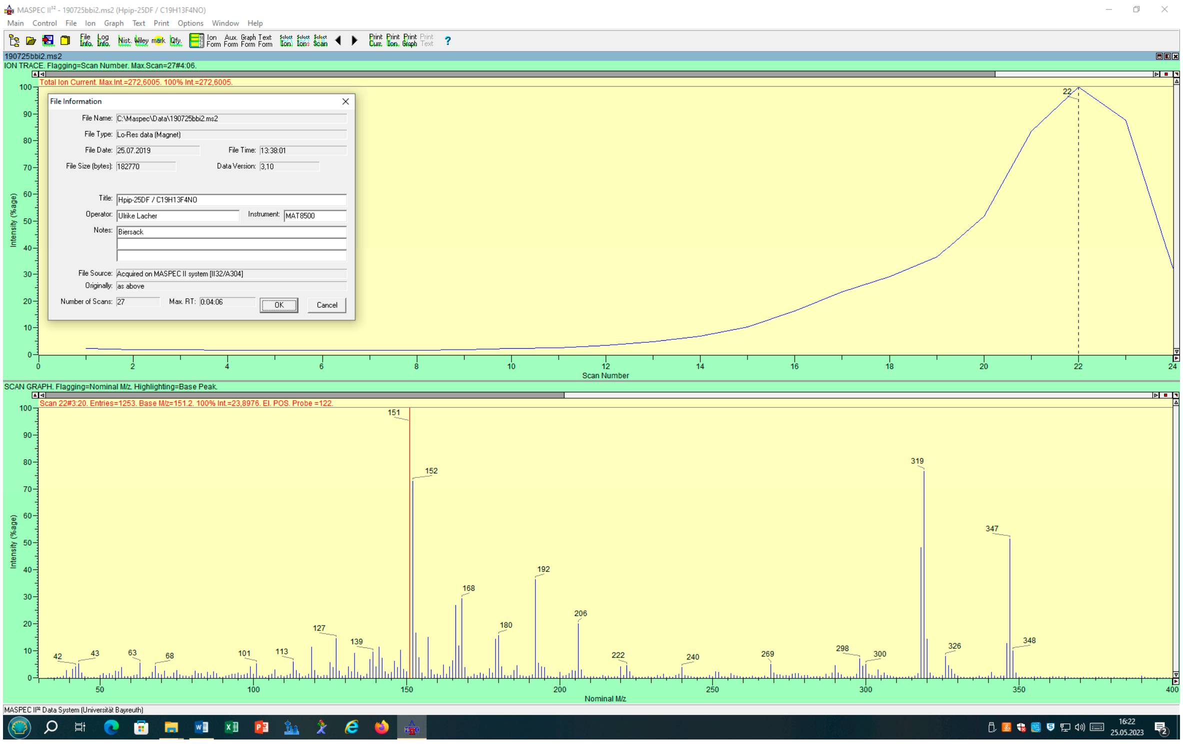

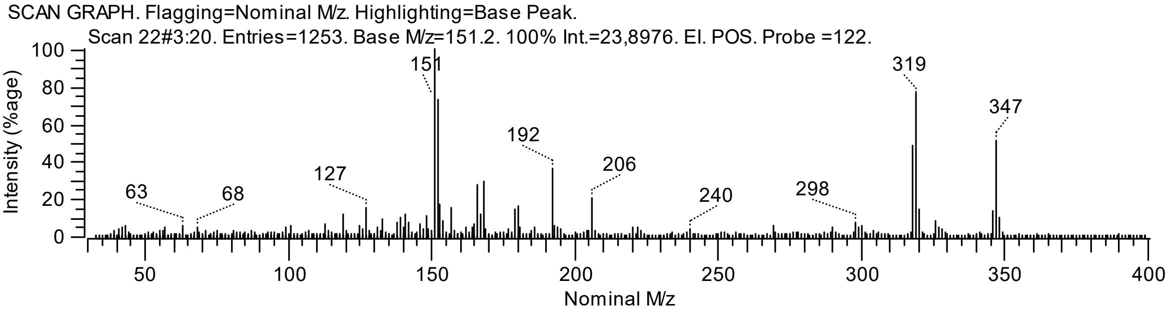

EI mass spectrum of **1g**

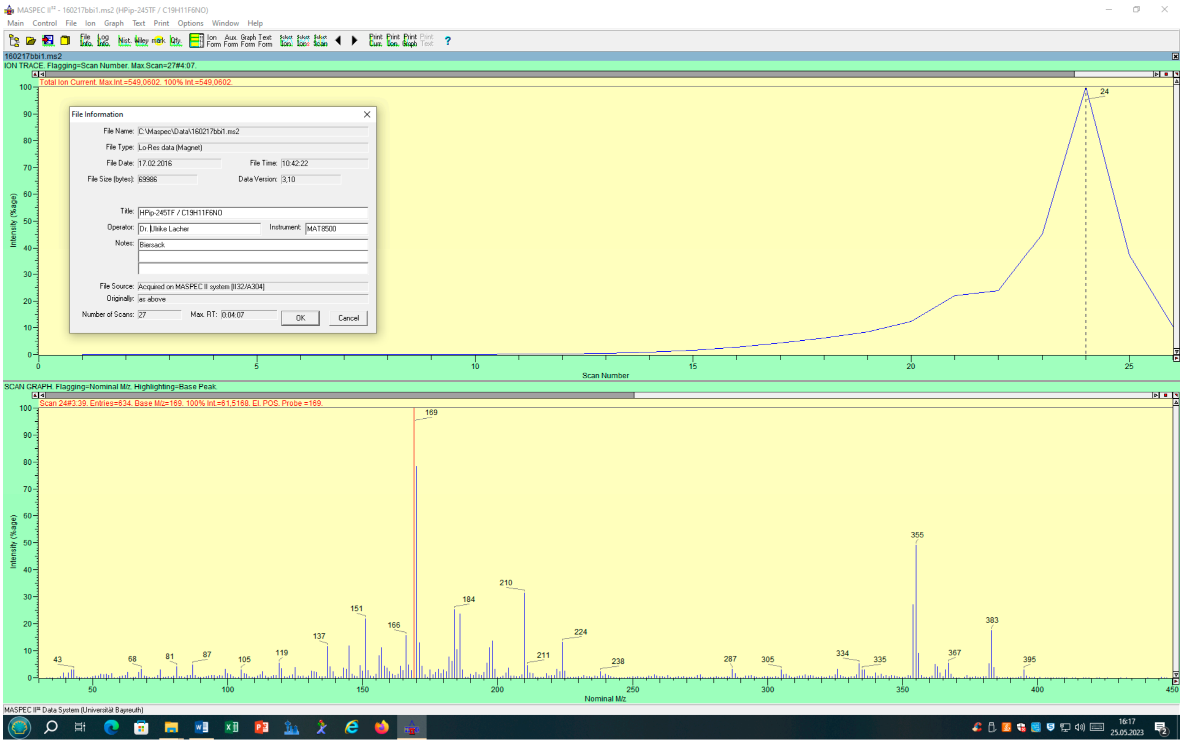

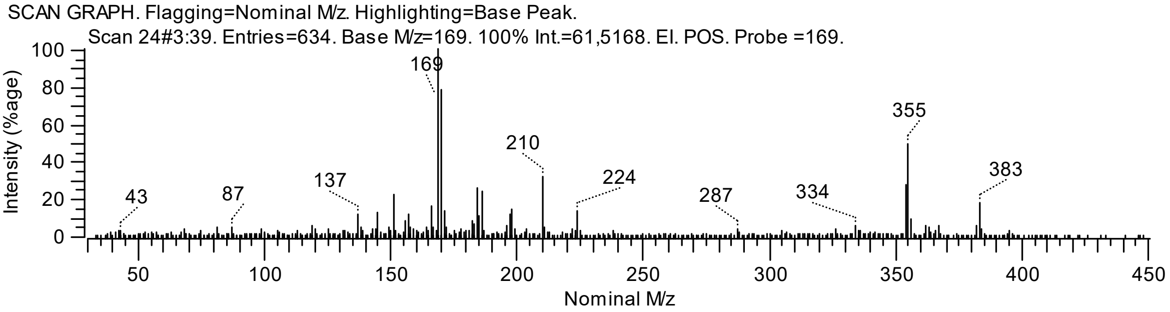

EI mass spectrum of **1h**

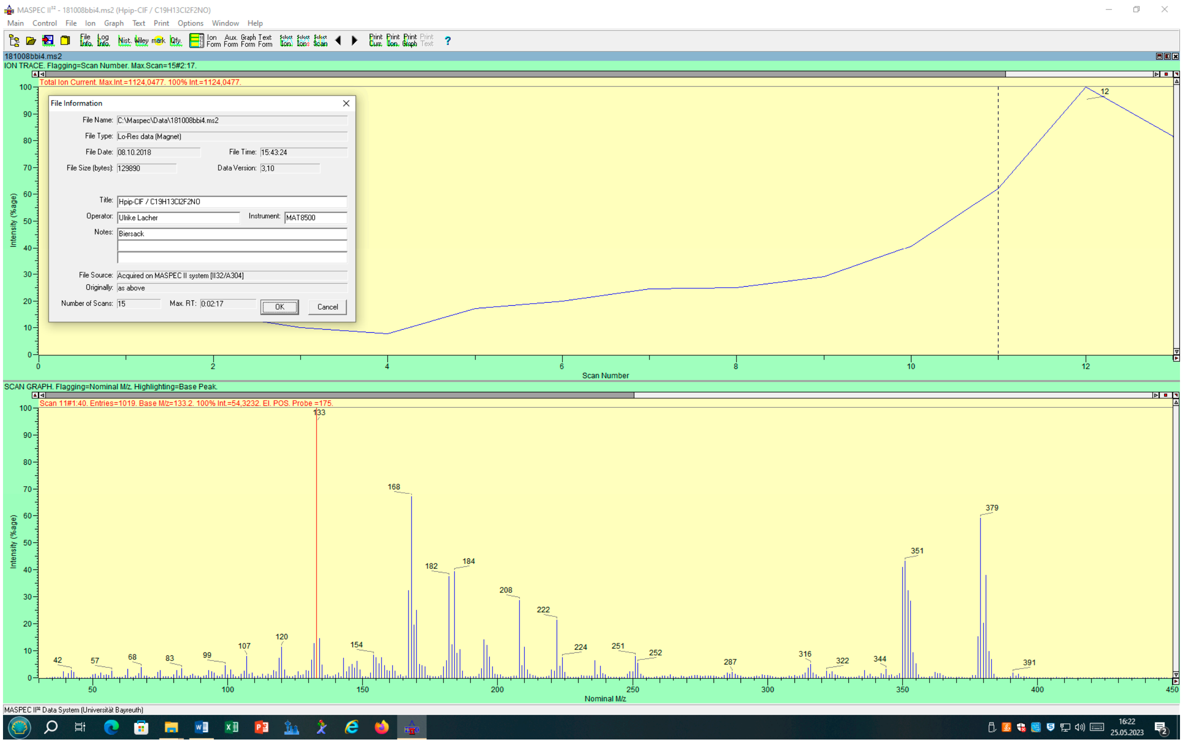

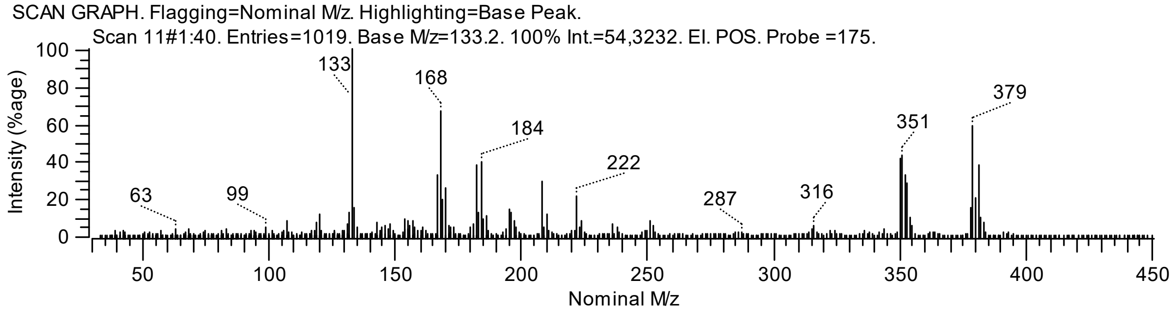

EI mass spectrum of **2c**

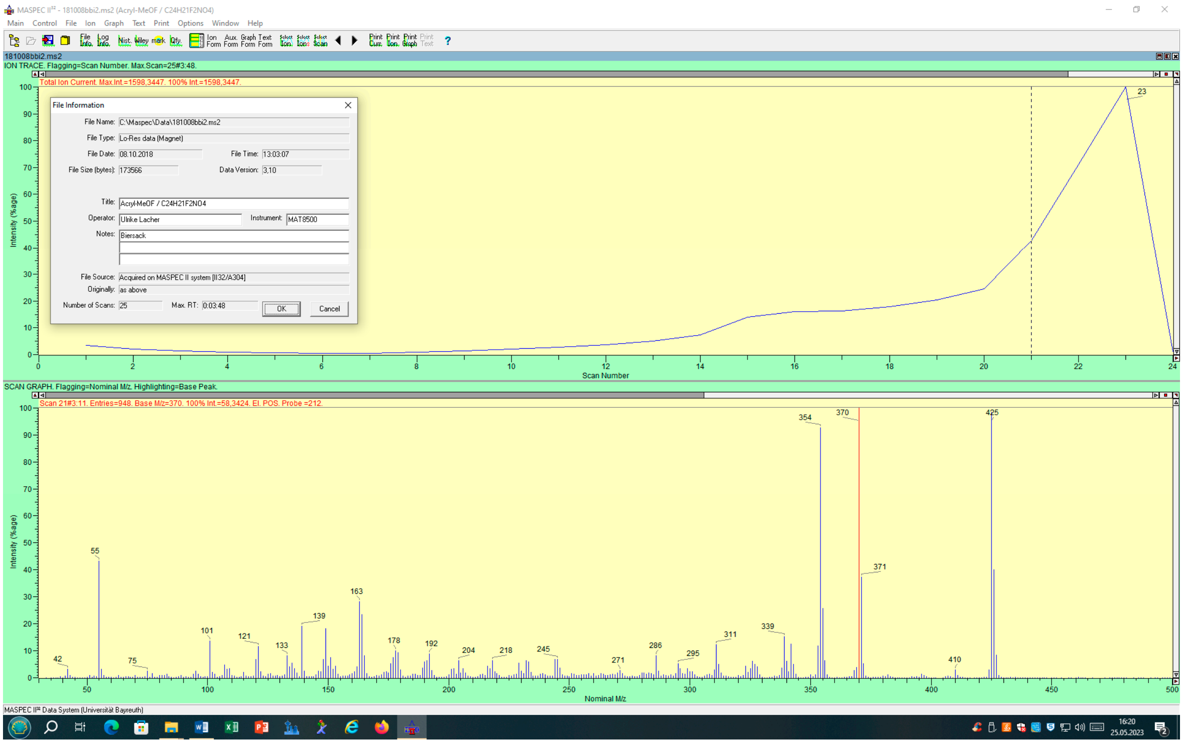

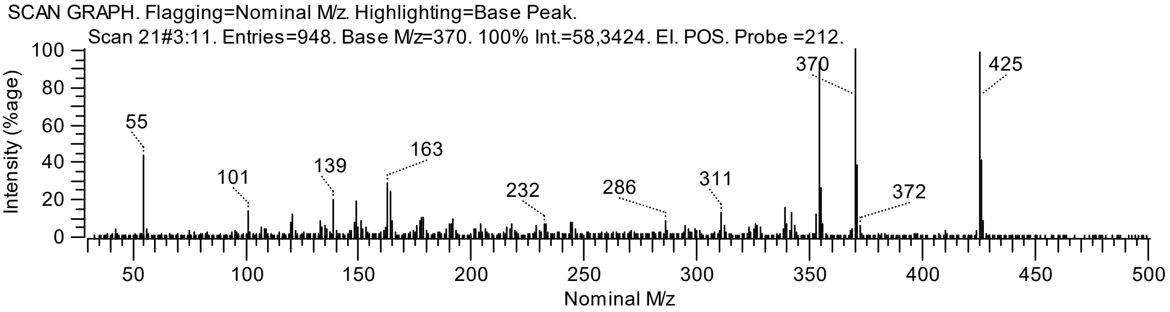

EI mass spectrum of **2e**

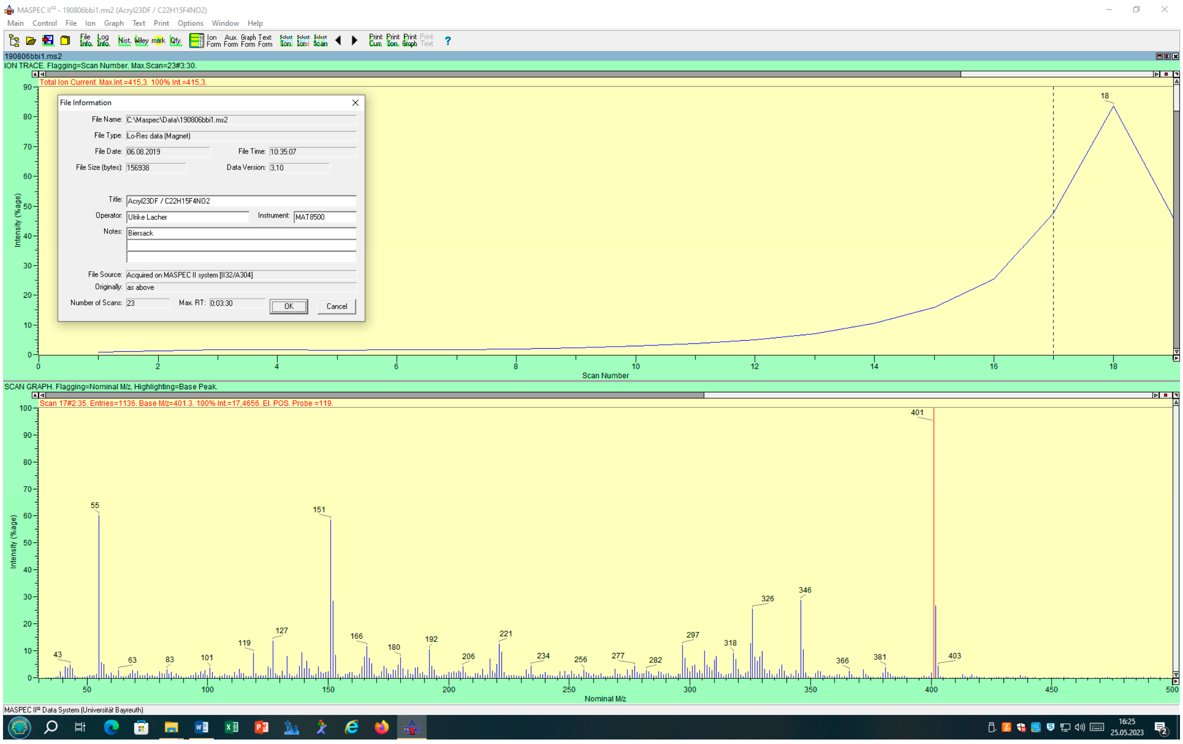

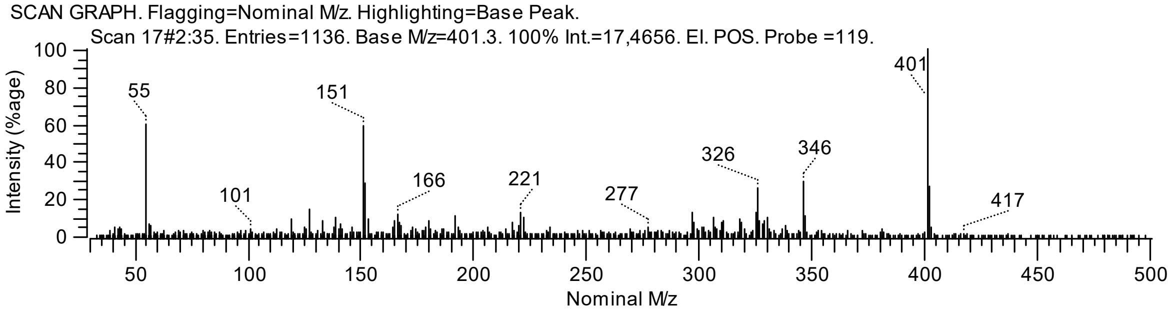

EI mass spectrum of **2f**

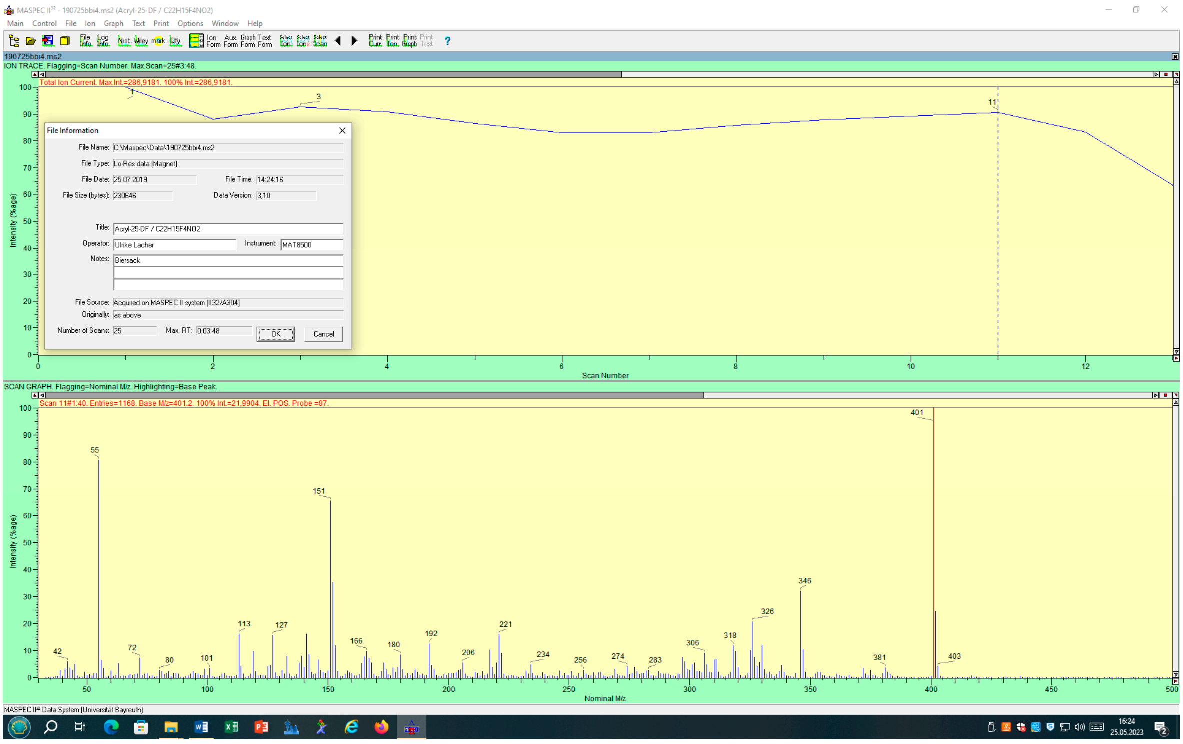

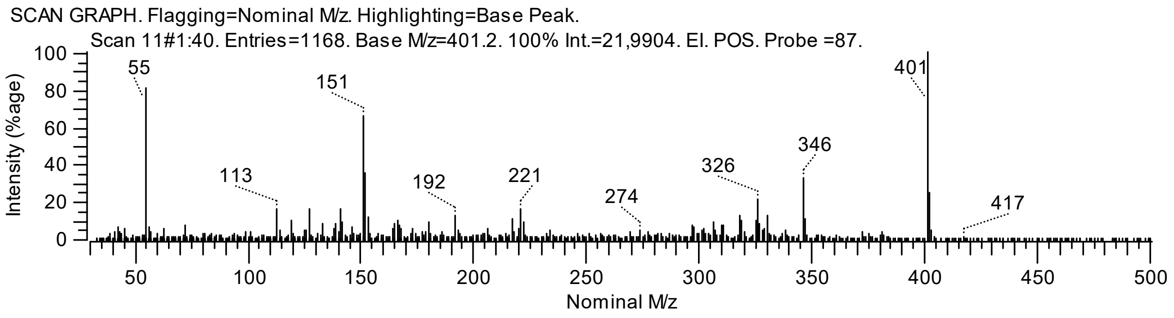

EI mass spectrum of **2g**

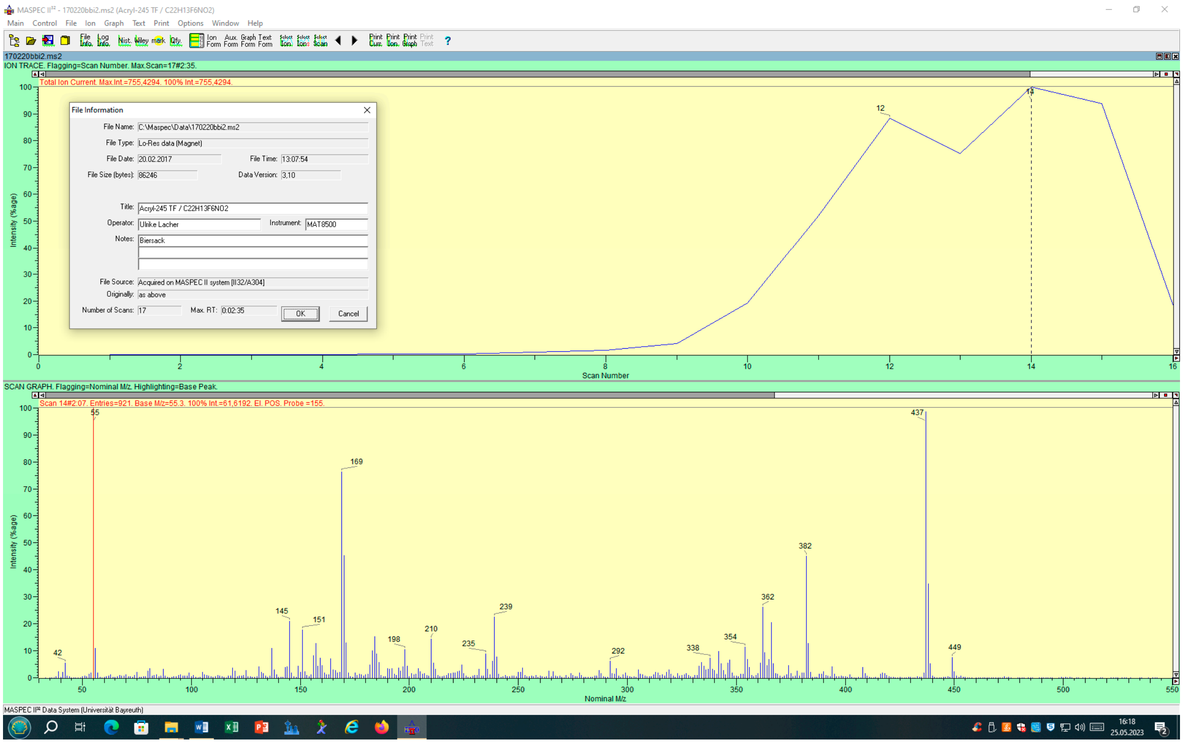

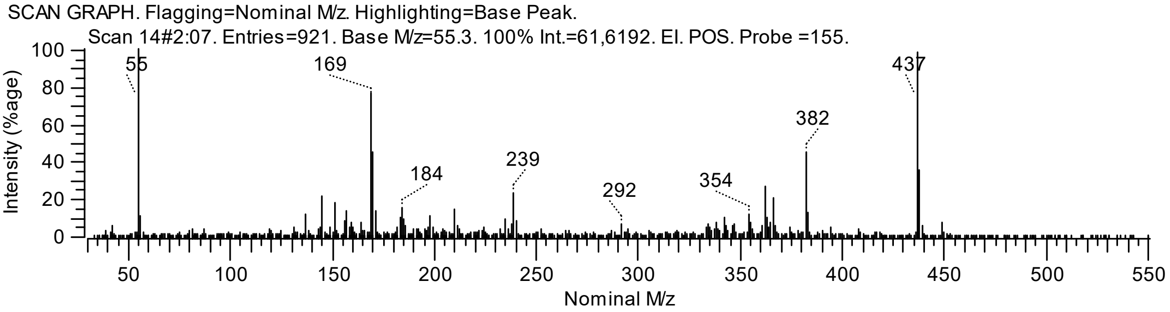

EI mass spectrum of **2h**

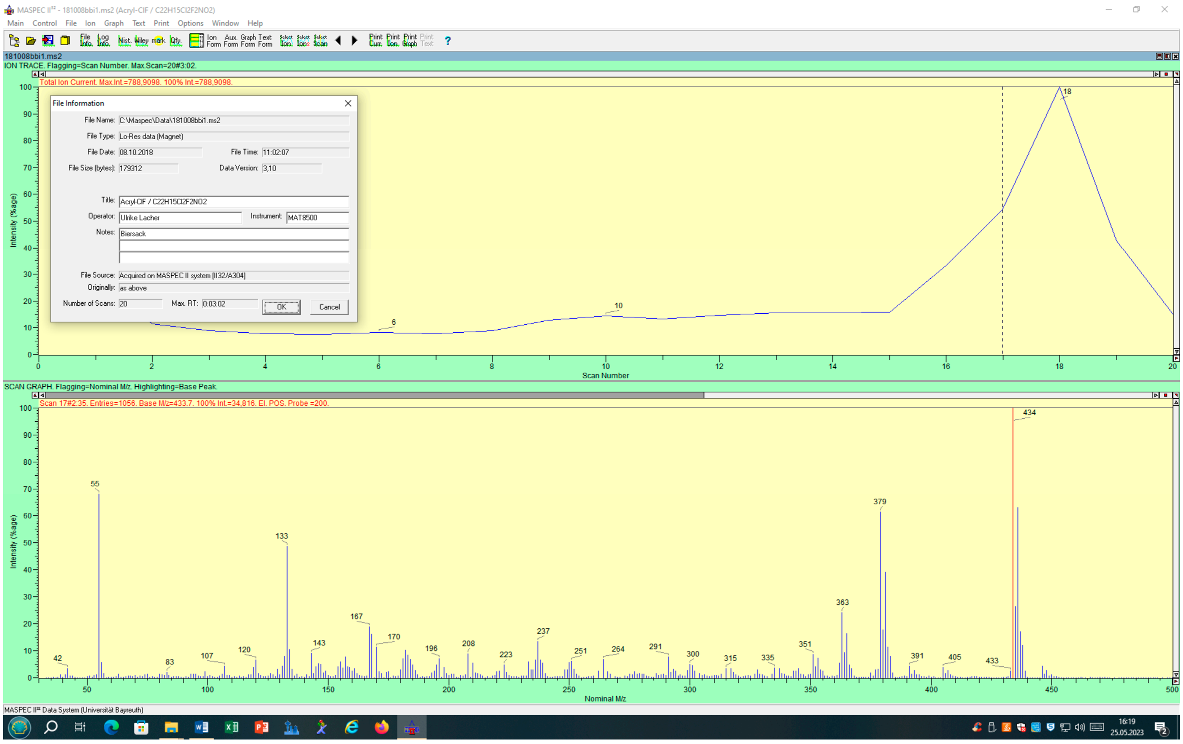

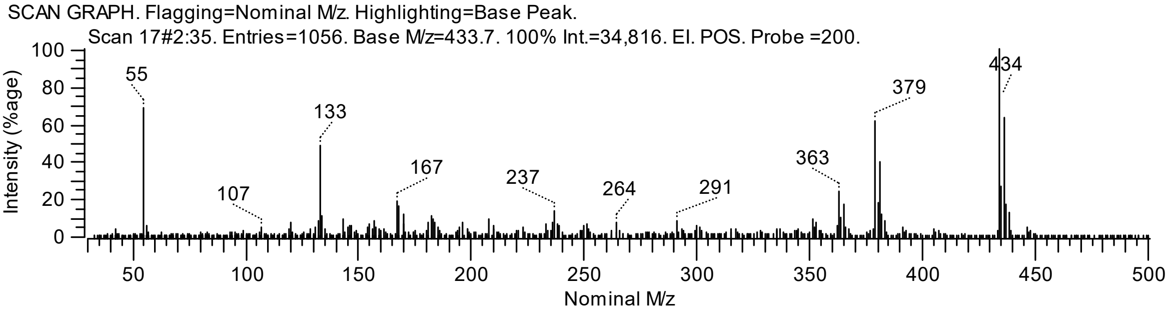

Supplement: Supplementary file 1 [file pharmaceutics-15-01921-s001.zip › pharmaceutics-2400305-supplementary.pdf]
